# The Peroxisome Proliferator-Activated Receptors of Ray-Finned Fish: Unique Structures, Elusive Functions

**DOI:** 10.3390/biom14060634

**Published:** 2024-05-29

**Authors:** Evridiki Boukouvala, Grigorios Krey

**Affiliations:** 1 Veterinary Research Institute, Hellenic Agricultural Organization-DIMITRA (ELGO-DIMITRA), 57001 Thermi, Thessaloniki, Greece; boukouvala@elgo.gr; 2 Fisheries Research Institute, Hellenic Agricultural Organization-DIMITRA (ELGO-DIMITRA), 64007 Nea Peramos, Kavala, Greece

**Keywords:** teleost fish, isotypes/isoforms, gene structure, functional domains, ligands/agonists

## Abstract

The Actinopterygian and specifically the Teleostean peroxisome proliferator-activated receptors (PPARs) present an impressive variability and complexity in their structures, both at the gene and protein levels. These structural differences may also reflect functional divergence from their mammalian homologs, or even between fish species. This review, taking advantage of the data generated from the whole-genome sequencing of several fish species, highlights the differences in the primary structure of the receptors, while discussing results from the literature pertaining to the functions of fish PPARs and their activation by natural and synthetic compounds.

## 1. Introduction

Interest in peroxisome proliferator-activated receptors (PPARs) in fish resulted from the need to address issues that were, and still are, of particular concern to the aquaculture industry. Specifically, the primary challenge encountered in the expansion of fin-fish aquaculture to an industrial-level activity concerned the sourcing of lipids for the diets of the farmed species. Aquaculture traditionally relied on fish meal and oil from industrial fisheries, a resource that is presently exploited at its maximum sustainable level. To secure its own sustainability, as well as to appease environmental concerns, the industry gradually conformed to the “less fish in than fish out” principle by developing and using aquafeeds that use less fish ingredients.

Efforts to replace fish oils with vegetable oils date more than 30 years [1]. However, vegetable oils possess distinct fatty acid profiles compared to fish oils, and, consequently, when included at high levels in the fish diets, they alter the flesh fatty acid composition; most importantly, they reduce the concentration of the n-3 long-chain polyunsaturated fatty acids eicosapentaenoic acid (EPA, 20:5n-3) and docosahexaenoic acid (DHA, 20:6n-3) that characterize the marine fish oil. DHA and EPA are associated with beneficial health effects, and are considered essential components in the human diet [2]. Fish oil replacement does not only affect the nutritional quality of the farmed fish but also compromises the health and well-being of the fish themselves [3]. The use of vegetable oil-based diets has been linked to liver and heart pathological conditions, immunosuppression, and impaired intestinal function [4,5,6]. These adverse effects may be exacerbated by additional stressors prevalent in contemporary aquaculture systems, including high stocking densities [7] and fluctuating environmental conditions [8]. To mitigate these effects, aquaculture-related research has invested a considerable effort to the exploration of lipid metabolism in farmed fish species, particularly at the gene/transcription level. In particular, PPARs have attracted considerable attention, due to their established roles in mammalian lipid metabolism [9], which followed the landmark cloning of a mammalian PPAR [10], the identification of three distinct PPAR isotypes (PPARα, β/δ, γ) from *Xenopus laevis* [11], and the recognition of fatty acids, eicosanoids, and various hypolipidemic agents as genuine ligands for these receptors [12,13]. Further interest for PPARs in aquaculture research stemmed from the findings that these receptors, and particularly the γ isotype, played a critical role in mammalian immune and inflammatory responses [14], since the industry suffers considerable loses due to bacterial, viral, and parasitic infections, as well as from other environmental factors that compromise the immune system of the fish.

Research on fish PPARs (herein referred to as fPPARs) was initiated by the cloning of the PPARγ isotype from both plaice (*Pleuronectes platessa*) and Atlantic salmon (*Salmo salar*) [15,16]. Subsequently, the presence of three PPAR isotypes was established in the European sea bass (*Dicentrarhus labrax*), as well as in gilthead seabream (*Sparus aurata*) and plaice [17,18]. To date, the cloning and characterization of fPPARs in over 30 fresh-water and marine fish species of particular interest to aquaculture is reported in the literature.

The interest in the study of fPPARs has not been limited to aquaculture-related issues. Fish species such as zebrafish have served as model organisms for studying human diseases associated with excessive adipogenesis [19], a process in which PPARs play a central role. Furthermore, the activation of PPARs by numerous industrial chemicals, including pharmaceuticals [20], has also been exploited in toxicological and environmental research for assessing the impact of these contaminants in aquatic ecosystems [21].

To date, the function of fPPARs on metabolism, affected by nutritional, ecotoxicological, inflammatory, or stress-related factors, has been studied in more than 50 fish species. This research involved the direct cloning of the cDNAs or genes for these receptors and, in addition to the insight into their functional properties, provided evidence of important structural differences between fPPARs and their homologues from terrestrial vertebrates, both at the gene and protein levels [18]. Furthermore, it revealed the existence of paralogues for each of the three receptor isotypes (see [22,23,24,25,26] for first reports of fPPAR paralogues). The existence of paralogous PPAR genes within the genomes of ray-finned fish (Actinopterygii, Teleostei) aligns with the hypothesis proposing an extra round of genome duplication (third round whole-genome duplication, 3R WGD) during the early evolution of this lineage [27,28,29] and suggests that the role of PPARs in fish physiology and biochemistry is more intricate compared to that in higher vertebrates.

The ever-increasing number of fish species with fully sequenced genomes provides the opportunity to further discern the structure of the genes encoding these receptors and speculate on the function of duplicated PPAR isoforms in the metabolic regulation of the fish species harboring them. Therefore, this review covers mostly the unique features and variety that characterize the structure of the fPPAR genes, and, consequently, of their encoded products. The current state of fPPAR research, concerning the functional properties of the receptors, and especially their activation by natural or synthetic compounds, is also discussed.

The new information resulting from the targeted cloning of fPPARs and/or from the whole-genome sequencing of different fish species underscores the need for a common nomenclature regarding the isoforms of the receptors. Indeed, it is often difficult for the reader of the relevant literature to understand, without resorting to a sequence homology analysis, which of the PPAR isotype–isoforms is being studied. To avoid further confusion, this review will follow the nomenclature used by GenBank [30] for the fPPAR genes and their protein products. Thus, the isoforms of each of the three PPAR isotypes will be distinguished by the letter a or b, following the gene/protein name (e.g., PPARαa, PPARαb). Consequently, the PPARβ/δ isotype will be referred to as PPARδ.

## 2. PPAR Isotypes and Isoforms in Fish Species: Diversity Reflects Complexity

Three distinct PPAR isotypes orthologous to the mammalian and amphibian counterparts, i.e., PPARα, δ, and γ, were first identified in the marine fish species European sea bass (*Dicentrarchus labrax*), plaice (*Pleuronectes platessa*), and gilthead sea bream (*Sparus aurata*) [17,18]. However, even at that time it was evident that this subfamily of nuclear receptors may present an increased level of complexity and diversity in teleost fish, owing to the teleost-specific extra round of whole-genome duplication (3WGD, or Ts3R) that occurred in the early evolution of this lineage. This was supported by the earlier identification of two different genes homologous to the mammalian PPARδ in the genome of zebra fish (*Danio rerio*) [22] and of two PPARα genes in the puffer fish (*Fugu rubripes*) genome [23]. Indeed, Leaver et al. [24] identified four different genes for the PPARδ subtype in Atlantic salmon (*Salmo salar*), of which at least two were shown to be functional, consistent with a fourth round of whole-genome duplication (4R WGD) inferred to the Salmoniformes lineage [31]. Subsequently, through gene/cDNA cloning, or through genomic analyses, two distinct isoforms of PPARα were identified in the turbot (*Scopthalmus maximus*) [32,33], the loach (*Misgurnus anguillicaudatus*) [34], the Japanese sea bass (*Lateolabrax japonicus*) [35], the brown trout (Salmo *trutta*) [36], the tambaqui (*Colossoma macropomum*) [37], and the cod (*Gadus morhua*) [38]. Similarly, two PPARδ isotypes were identified in the tambaqui [37] and the loach [39]. The duplication of the PPARγ gene in a teleost fish was first established in the blind cave fish (*Astyanax mexicanus*) [26], while more recently through comparative genomic and phylogenetic analyses, [40] two PPARγ genes were identified in the genomes of European sardine, *Sardina pilchardus* (Clupeiformes), herring, *Clupea harengus* (Clupeiformes), red-bellied piranha, *Pygocentrus nattereri* (Characiformes), northern pike, *Esox lucius* (Esociformes), and channel catfish *Ictalurus punctatus* (Siluriformes), as well as in the salmonid species Atlantic salmon, Coho salmon (*Oncorynchus kisutch*), and rainbow trout (*Oncorynchus mykiss*).

For the purposes of this review, a non-exhaustive search of teleost fish species with sequenced genomes revealed that two PPARα paralogues are present in 34 out of the 40 different Orders examined (178 species, Table S1). According to this search, the PPARαa isoform has not as yet been identified in the Acipenseriformes, Gonorynchiformes, Osmeriformes, and Osteoglossiformes Orders, while in the Blenniiformes and Holocentriformes, it is the αb isoform that is presently missing. In contrast, PPARδ duplicates were identified in only 9 out of the 33 Orders examined (150 species) and specifically in the Anguilliformes, Characiformes, Clupeiformes, Cypriniformes, Esociformes, Gymnotiformes, Osmeriformes, Salmoniformes, and Siluriformes (Table S1). Similarly, the search for PPARγ paralogues in 41 teleost Orders (173 species) identified, in addition to the species mentioned by [40], a duplicate gene in the Osmeriform species *Osmerus eperlanus* (GenBank Access. nos. XM_062459215 and XM_062459587, Table S1). It is of interest to note that the species that retain gene duplicates for both PPARδ and γ do not belong to Neoteleostean Orders. Specifically, and according to [29], the Characiformes, Clupeiformes, and Siluriformes are phylogenetically placed in the Otocephala branch of Clupeocephala, while the Salmoniformes, Esociformes, and Osmeriformes are placed in, as yet, unnamed Euteleostean clades that diverged prior to the Neoteleosteans. As both Cypriniformes and Gymnotiformes are placed in the Otocepha branch, it is reasonable to expect that PPARγ duplicate genes will be identified in the species of these Orders. It remains, however, to be confirmed whether the presence of PPARδ and PPARγ duplicates will remain limited to only these Teleostean Orders, or will be identified in additional ones, as the assembly and annotation of fish genomes progresses.

Deciphering the potential functional divergence of the duplicated loci of the three fPPAR isotypes is a fundamental issue to our understanding of the biology of these receptors in fish species.

## 3. The Functional Domains of fPPARs

The proteins encoded by the fPPAR genes, just as their homologues from higher vertebrates, are organized in four functional domains: the A/B domain, located at the NH_2_-terminus of the protein; the C or DNA-binding domain; the D domain or hinge region; and the E domain or ligand binding domain.

### 3.1. The A/B Domain

In mammals, the A/B domain contains activation function 1 (AF1) that has been shown to modulate ligand-independent PPAR activity via phosphorylation [41,42] and is encoded by a single exon, except in the case of alternative splicing, which, as in the case of the mammalian PPARγ isotype [43,44], results in a short and long A/B domain and, consequently, in a short and long receptor form (PPARγ1 and PPARγ2, respectively). In Teleost fish species, this domain is also encoded by a single exon, as splice variants have not as yet been formally identified. The function of this domain remains unexplored in the fish receptors, despite the fact that it presents interesting structural features that will be discussed below.

The examination of the protein sequence of the A/B domain in the two PPARα isoforms from the species listed in Table S1 revealed that the length of this domain ranges from 57 to 75 amino acid (aa) residues, as compared to 65 aa in the human receptor, with 57 aa being the length encountered in 70% of the species examined. In these species, the initiation methionine is followed by Ala-Gly-Asp-Leu (MAGDL motif), while in the longer-length sequences, the MVDM motif is found, as is also the case in the mammalian receptor.

In the PPARαb isoform, the A/B domain length ranges from 59 to 108 aa, being 65 aa long in 70% of the sequences examined. In all the PPARαb sequences, the MVDX motif is found, where X is an aliphatic residue. In the species with duplicate PPARα genes, the two isoforms share 40–50% sequence similarity in this domain. Notable is the low degree of similarity between isoforms in the species of the Tetraodontiformes, Siluriformes, and Gadiformes Orders (≈25%), as well as the high degree of homology (≈80%) in the two Atlantic salmon isoforms.

The PPARδa A/B domain, in the few species where this isoform has been identified, has an average length of 118 aa. Sequence identity between species is relatively low, averaging 27%.

In the PPARδb isoform and in the sequences examined, the domain length has an average length of 113 aa, but is considerably longer in the Acipeceriformes (one species, 175 aa), Clupeiformes (five species, 150–200 aa), and Polypteriformes (one species, 186 aa). Sequence identity between species, in 25 of the 35 Orders examined, is relatively high, ranging between 40 and 70%. The Clupeiformes, Cypriniformes, Esociformes, Gadiformes, Gymnotiformes, Osmeriformes, Osteoglossiformes, Salmoniformes, and Siluriformes sequences diverge considerably, exhibiting low levels of sequence identity with the other Orders or between them (<17%). As expected, the most distantly related between all sequences are those from the Acipenseriformes and Polypteriformes species. The within-species sequence identity between the two PPARδ isoforms is approximately 15%.

The fPPARγ A/B domain also varies considerably in length and, according to the sequences examined (Table S1), it ranges from 76 aa in the sterlet (*Acipenser ruthenus*, Acipenceriformes) to 332 aa in the chum salmon (*Oncorynchus keta*, Salmoniformes). The MVDT motif is present in the vast majority of the sequences examined, although it is, in most of them, preceded by a sequence containing the motif MQTP. As most of these sequences are predicted from genome analyses and have not been functionally characterized, it remains to be demonstrated which of the two motifs marks the translation initiation site, considering that the sequences encoding the two motifs are located in different and distal (>20 kb) exons (e.g., see GenBank entries XM_026350118.2 and XM_023954652.1, among others), with the upstream exon encoding only for 19 aa.

In the species with duplicate genes, moderate sequence identity is observed in this domain (33–66%) for the, tentatively labelled, PPARγa isoform between different Orders. In contrast, for the PPARγb, identity is low (<23%). It is of interest to note that in the chum salmon, there are apparently two PPARγb isoforms (GenBank: XM_052481996 and XM_052483128). Both isoforms have an A/B domain extremely rich in histidine residues, which is also characterized by the presence of multiple repeat sequences. Thus, in the PPARγb1 sequence (XM_052481996), the sequence NHSFNHPDI/R is repeated 28 times. In the PPARγb2, the sequence SHNSPHHH is tandemly repeated 11 times, while there are an additional 11 tandem repeats of the sequence SPDRSHSFNH. The rainbow trout PPARγb A/B domain is also His-rich and contains 22 tandem repeats of the SPDRSHSFNH sequence, while it has only one copy of the SHNSPHHH motif. Repeated elements have also been identified in the PPARγa isoform of the channel catfish (GenBank: XM_017450361.3), where 16 copies of the sequence QENSYRA are found. Similarly, in the northern pike PPARγa (GenBank: XM_034297489.1), 11 tandem repeats of the QPSSSLPQHH motif are present. Repeated motifs have also been reported in the Atlantic salmon A/B domain [16,45] as well as in that of the yellow catfish [46]. It has been proposed that these repeated motifs may function in coactivator recruitment [45].

### 3.2. The DNA-Binding Domain

The DNA-binding or C domain (DBD herein) is the most conserved region of the nuclear hormone receptors and is responsible for the recognition and binding to specific response elements in the promoters of genes regulated by these transcription factors [47,48]. As is the case in higher vertebrates, the fPAAR domain is encoded by two different exons, one for each of the two zinc fingers of the domain. All fPPAR sequences maintain the characteristic D box structure, containing three instead of five amino acids between the first two Cys residues of the second Zn finger [11].

In the PPARαa isoform, the two exons encode a sequence that ranges in length from 94 aa in the Salmoniformes to 106 aa in the Gadiformes, with 102 aa being the length encountered in the majority of sequences examined (>60%). Sequence identity between species from different Orders ranges from 67 to 94%, with the large variation being due to the sequence upstream of the first Zn finger. When just the core of the domain is considered, i.e., excluding the upstream to the first Zn finger sequences, the identity is greater than 87%, reaching 100% in some cases.

For the PPARαb isoform, the most frequently encountered domain length is of 105 aa (>50% of the sequences examined), followed by domain lengths from 102 to 104 aa. Sequence identity of the core domain between species from different Orders ranges from 90 to 100% for this isoform. In the species with duplicate genes for PPARα, sequence identity between isoforms ranges from a low of 70% in the Gadiformes (86% in the core) to a high of 91% (99% in the core) in Salmoniformes. Notable is also the high sequence identity (84%, 97% in the core) between the two isoforms in the Anguilliformes.

The DBD of the PPARδa sequences examined varies in length between 93 and 102 aa. The sequence identity between species of different Orders for the full domain is between 67 and 87%, while at the core of the domain, it is between 88 and 97%. In contrast, in the PPARδb sequences, the domain length presents a more restricted variability with 99 aa in 48% of the sequences and 97 aa in 42% of them, with the remaining having either 96 or 98 aa. Sequence identity in the full domain ranges from 71 to 99% (85 to 100% in the core). In the species retaining the two PPARδ loci, sequence identity between isoforms ranges in the full domain, from a low of 72% between the Cyprinoformes (88% in the core) to a high of 84% in the Characiformes and Osmeriformes (100% in the core).

The PPARγa DBD in the species examined varies in length from 101 to 105 aa, with the 101 aa and 102 aa lengths representing 61% and 28% of the sequences, respectively. Sequence identity between species for the full domain is 80% on average, while at the core, it is 98%.

The Clupeiformes, Siluriformes, and Characiformes PPARγb domain has a length of 102 aa; of the Salmoniformes, 108 aa; of the Esociformes, 111 aa; and of Osmeriformes, 117 aa. Sequence identity between species is 63% on average in the full domain and 82% at its core. Within species, sequence identity between isoforms is 72% for the full domain in the Clupeiformes and Esociformes, 70% in the Salmoniformes, 65% in the Osmeriformes, 61% in the Characiformes, and 59% in the Siluriformes. At the core of the domain, the identity is 85%, 97%, 92%, 85%, 78%, and 72%, respectively.

Residue deletions or insertions, relative to the corresponding human PPAR sequences, in all isoforms concern the region upstream of the first Zn finger and do not affect the Zn fingers’ structure or the spacer between them.

PPARs bind as heterodimers with a retinoid X receptor (RXR) on response elements (PPREs) of the consensus sequence AGGTCA N AGGTCA (DR1-type element) [49], with residues upstream of the core element determining the polarity of the PPAR:RXR heterodimer [50]. In vitro, by means of the electrophoretic mobility shift assay (EMSA), the binding of European sea bass, gilthead sea bream, and plaice PPARαa, δa, and γa isotypes, as heterodimers with the mouse RXRβ, has been demonstrated on well-characterized mammalian PPREs (ACOx, Cyp4A6z) and on potential elements present in the plaice glutathione-S transferase A1 gene (*gsta1*) promoter [17,18]. In transient transfection experiments, PPAR holoproteins of the same species were able to drive transcription through a synthetic promoter containing the mouse Cyp4A6z PPRE [18]. The same promoter was also used to demonstrate the transactivation potential of the Atlantic salmon PPARδ isoforms, while a promoter containing the mouse ACOx PPRE was used for PPAR-dependent transcription by the torafugu pufferfish receptors [25]. Although putative PPREs have been identified in the promoters of the zebrafish fatty acid-binding protein (FABP) genes [51,52,53], as well as in the Δ5Δ6 fatty acid desaturase 2 (*fads2*) promoter of the rabbitfish (*Siganus canaliculatus*) [54], these elements have not been tested for their functionality in in vitro (EMSA/transactivation) assays. Point mutations on the putative PPRE identified in the promoter of the yellow catfish (*Pelteobagrus fulvidraco*) PPARα gene downregulated, but not abolished, both the basal and fenofibrate-induced expression of the reporter gene in transient transfection experiments [55]. According to the above and due to the high conservation of the DBD and the overall structure of the proteins among species and lineages, it is expected that fPPARs can recognize and bind to canonical DR1 elements. However, the study of the promoters of putative PPAR target genes in fish species and the potential receptor–DNA interactions on native promoters merits more attention.

### 3.3. The D Domain (Hinge Region)

The D domain, or hinge region, located between the DNA-binding and ligand binding domains, has been proposed to condition DNA-binding affinity of the receptors by interactions with DNA sequences upstream of the response element [50,56]. The domain in fish, as in higher vertebrates, is encoded by a single exon that also includes helices 1 and 2 (H1 and H2), which historically have been considered as part of the ligand binding domain of the receptors (see also below).

In PPARαa, the encoded sequence of the exon has a length of 67–69 aa, and an average between-species identity of approximately 70%. In the PPARαb, it has the invariable length of 67aa in all the sequences examined and an average identity of 84% between species. Between isoforms and within species, sequence identity is lower to that of the between-species comparison, ranging from 51% in Gadiformes to 77% in Lampriformes.

For PPARδa, the encoded sequence has a length of 68 aa in the majority of species examined, with the remaining having 69 aa. A longer domain (74 aa) is found in the Atlantic salmon β1A sequence (GenBank: NM_001123635). Between species, sequence identity averages 76%.

For PPARδb, the sequence length in the vast majority of the species (>80%) is 68 aa, with the remaining ranging from 64 to 69 aa. Between species, sequence identity averages 83%. Within species, sequence identity between isoforms is 82% in the Anguilliformes (1 species, European eel, *Anguilla anguilla*), 79% in the Clupeiformes, 74% in the Gymnotiformes (1 species, electric eel, *Electrophorus electricus*), 71% in the Salmoniformes (1 species, Atlantic salmon), and 69% in both Characiformes and Esociformes.

For PPARγ and in species of Orders with a single gene for the receptor, i.e., the tentatively labelled PPARγa isoform, the length of the encoded sequence is invariably 67 aa and exhibits a high degree of identity (>90%) between species. For the PPARγb isoform, the domain has 65 aa in the northern pike; 66 aa in the salmonoid species, with the exception of Atlantic salmon (67 aa); and 67 aa in the Characiformes, Clupeiformes, and Siluriformes species. Between species, sequence identity is 55% on average, while within species, the sequence identity is slightly higher, at 57% on average.

Residue deletions or insertions, relative to the corresponding human PPAR sequences encoded by this exon, occur upstream of H1 of the LBD. While the sequence of H2 is highly conserved between the fish and the human receptors, the sequence corresponding to H1 diverges considerably but still retains helical properties, as suggested by protein structure prediction models [46].

### 3.4. The Ligand Binding Domain

The PPAR ligand binding domain (LBD herein) is the most interesting domain of the receptors due to its unique structural characteristics. This domain, which assumes a similar structure in all three isotypes and resembles that of the other nuclear receptors, contains 13 helices and a small four-stranded β-sheet. Therefore, compared to the other nuclear receptors, the PPAR LBD contains an extra helix, located between the first β-strand and H3, which, according to the nomenclature proposed by Uppenberg et al. [57], is termed H2B or H2’ [58]. The LBD, in addition to ligand binding, provides the dimerization interface with RXR [59], as well as the ligand-dependent activation function 2 (AF-2), located at the C-terminus of the domain in H12, which is responsible for co-activator recruitment and binding. Characteristic of the PPAR LBDs is the size of the ligand cavity that is considerably larger as compared to other nuclear receptors. The ligand binding cavity is Y-shaped and is divided into two arms: Arm I that reaches towards H12 (AF-2) and Arm II that is situated between the β-sheet and H3 [58]. At the entrance of the binding site, a loop is formed between helices H2’ and H3, the so-called ω-loop, which due to its flexibility, may allow molecules of different sizes to enter the pocket. Both the large size of the binding pocket and the presence of the ω-loop may explain the multi-ligand activation property of PPARs. Although the crystal structure of a fish PPAR has not as yet been determined, structural models suggest that the fPPAR LBDs retain the overall structure of the mammalian receptors with unique, however, structural and potentially functional, features.

As previously mentioned, in the fPPARs, and as is the case for the amphibian and mammalian receptors [60,61], helices 1 and 2 of the LBD are encoded by the exon that also contains the hinge region (D domain) sequences. The remaining structural features of the domain, starting from the first β-strand of the β-sheet, are encoded by two exons in higher vertebrates. In contrast, the initially characterized genes for the PPAR isotypes from European sea bass, gilthead sea bream, plaice, and Atlantic salmon [17,18,24] revealed that in these fish species, the region corresponding to the first exon of the mammalian LBD is encoded by two exons in the α and δ isotypes (αa and δb, according to the nomenclature used herein) and by three exons in the γ isotype, resulting in “three and four exon LBDs”, respectively. Unexpectedly, and according to genome sequencing data, this structural organization is not always observed in the receptors of the different Teleost Orders.

#### 3.4.1. The fPPARα LBD

**Figure 1:**
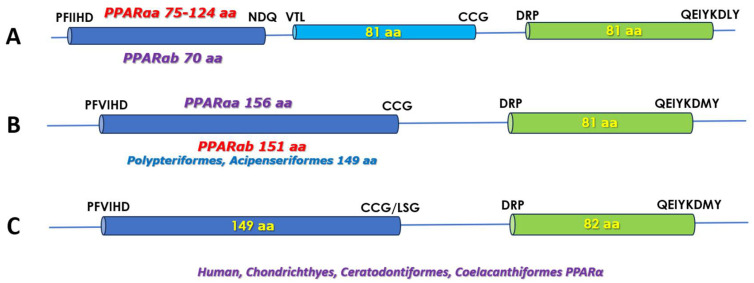
A schematic representation of the exons encoding the LBD of the fPPARα isoforms. (**A**) The 3-exon LBD type for the PPARαa (red) and PPARαb (mauve) isoforms. (**B**) The 2-exon LBD type for the PPARαa (mauve) and PPARαb (red) isoforms. (**C**) The LBD exons in the mammalian and non-Actinopterygian fish PPARα. Residues encoded by each exon are indicated above or on the exons. The aa sequences at the exon boundaries are also shown.

As depicted in Figure 1, the two isoforms of the PPARα isotype are encoded by genes that have distinct structure as far as the number of the LBD exons is concerned. Accordingly, the isoforms can be divided into two distinct groups.

The first group (group I) refers to species in which the LBD is encoded by three exons in the PPARαa and by two exons in the PPARαb. This arrangement is encountered in the majority of Orders/species in which duplicate PPARα genes have been identified.

In these species, the encoded LBD sequence ranges from 233 to 286 aa in the PPARαa isoform (Figure 1A) and is, thus, considerably longer than its mammalian counterpart (231 aa). This is due to the first exon, which contains an extended ω-loop, characteristic of the PPARα and γ isotypes [18]. The most extended ω-loop was observed in the Gobeiformes species *Boleophthalmus pectinirostris* and *Periophthalmus magnuspinnatus* (57 and 48 aa, respectively) and the shortest in the Anguilliformes species examined (3 aa). The ω-loop in the “group I” Orders/species contains variations (1–10 aa substitutions and/or additions or deletions of flanking residues) of the sequence PEPESGLQAGEXVPAVGCG. In most cases, the loop is preceded by an acidic residue and follows an LQ motif. Between species in different Orders, the overall LBD sequence identity is ≈90% for this isoform. It is noted that in the third exon, and in the spacer sequence between H11 and H12, the isoform suffers a one-residue deletion, as compared to the human PPARα (Ser 452).

In “group I” of Teleost species, the PPARαb isoform LBD is encoded by two exons, the first of which corresponds to exons 1 and 2 of PPARαa. The domain of this isoform has an invariable length of 232 aa, and, consequently, as compared to the mammalian LBD, the ω-loop is extended by two aa residues. However, in the sturgeons and bichirs where only the PPARαb isoform has thus far been identified, the first exon encodes a 149 aa sequence, as is the case in the mammalian, lungfish, coelacanth, and chondrichthyan receptor. As is the case for the αa isoform, the one-residue deletion, at the equivalent of position Ser 452 in the human receptor, is also observed in these sequences. Sequence identity of the LBD of the PPARαb isoform between the different species is >90%. Between the two isoforms, identity in this domain does not exceed 80%, with the ω-loop not affecting this score significantly.

In the second group (“group II”) of species that includes those of the Characiformes, Clupeiformes, Cypriniformes, Gymnotiformes, and Siluriformes Orders, the sequences listed in GenBank as the PPARαa isoform have two exons encoding the LBD, while three exons encode the domain in those predicted as the PPARαb isoform. According to the available sequence data, the two LBD exons in PPARαa encode invariably for 156 and 81 aa sequences, with the intron/exon boundaries positioned exactly as in the two-exon PPARαb LBD (Figure 1B). The ω-loop in this isoform type is extended by 7 aa residues as compared to the human receptor. The loop sequence is not conserved between species of different Orders but is highly conserved in species of the same Order.

In the “group II” species, the LBD of the αb isoform has an invariable length of 232 aa with the exon/intron boundaries positioned exactly as in the 3-exon PPARαa type (Figure 1A). The sequence between species for this isoform is also highly conserved (>90%) and the within-species sequence identity of the two isoforms exceeds 75%. It is of interest to note that the 2-exon PPARαa LBD sequences are more closely related to the 2-exon PPARαb LBD ones, than to those of the 3-exon PPARαa, implying that their classification as the PPARαb isoform possibly needs reconsideration. In both of these isoforms, the one-residue deletion at the terminal LBD exon is observed, as is the case in the “group I” receptors.

Note, also, that the ancient non-ray-fin fish lineages of Chondrichthyans (chimaeras, rays, skates, and sharks), Coelacanthiformes (coelacanths), and Ceratodontiformes (lungfish), which diverged from the Actinopterygii before the 3R WGD, share the same gene structure and LBD length with the mammalian receptor (Figure 1C). In the also ancient Actinopterygian, but not Teleostean, Orders of Polypteriformes (bichirs) and Acipenseriformes (sturgeons), the sequence encoded by the first LBD exon is of the same length as that of the mammalian receptor (149 aa, Figure 1B), i.e., it lacks an extended ω-loop sequence. However, in both Orders, the one-residue deletion in the second exon is observed. Therefore, it appears that the ω-loop extension event in the fPPARα coincides with the evolution of the Teleostean lineage about 350 million years ago. Also to note is the fact that all “group II” Orders, i.e., Characiformes, Clupeiformes, Cypriniformes, Gymnotiformes, and Siluriformes, phylogenetically resolve in the Otocephala clade that diverged from the Euteleosteans, to which the majority of the other Orders examined belong, about 270 million years ago [29,40].

Concerning the aa residues identified as critical for ligand binding to the receptor (reviewed in [58]), the majority of them are conserved in the fPPARα isoforms (Table 1), with most substitutions being conservative, except for two residues in the entrance sequence of the binding site. There, at the equivalent position of V324, a Cys is present in the PPARαa isoform and the Y334 is substituted by the positively charged Arg, as is also the case in some PPARαb sequences. Furthermore, a degree of variability is observed between the sequences of the fish receptors, especially in PPARαb, with potentially important consequences in ligand binding (e.g., substitution of Y314 by His, or Arg instead of Glu at position 251 in the Arm I and Arm II sites, respectively). However, these need further confirmation as they may represent sequencing errors.

Evidence for direct binding of ligands to the fPPARα LBD is limited to the European sea bass αa isoform and includes natural fatty acids, as well as the specific mammalian PPARα ligands pirinixic acid (Wy-14643) and 5,8,11,14-eicosatetraynoic acid (ETYA) [17]. The recognition of these compounds by the fish receptor is in accordance with the positional conservation of the Arm I residues of the binding pocket (Table 1). Through transient transfection experiments with the native plaice and seabream PPARαa in sea bass larval cells, Leaver et al. [18] demonstrated that the receptor is activated by a variety of natural fatty acids, as well as by Wy-14643 and ETYA, but not by the known activator of mammalian PPARα, and environmental obesogen, perfluoroctanic acid (PFOA) [62]. The receptor was also unresponsive to the mammalian PPARγ-specific ligands rosiglitazone and GW1929. Similarly, using both PPARα isoforms from the torafugu pufferfish for transfections in a goldfish cell line, Kondo et al. [25] also demonstrated the activation of the receptors by fatty acids, Wy-14643, and ETYA and, furthermore, showed a small but significant activation of PPARαb by rosiglitazone. Similar results were also reported for the medaka (*Oryzias latipes*) PPARαa [63].

To maximize the transfection assay specificity, Colliar et al. [64] used the plaice PPARαa LBD fused to the Gal4 DNA-binding domain in flathead minnow cells. In addition to confirming previous results, they showed a strong response of the receptor to bromopalmitate, gemfibrozil, and the mammalian PPARδ-specific ligand GW501506, as well as to the mono-1methyl-hexyl-phthalate. As in the previous study, PFOA failed to induce transcriptional activity of PPARαa. In the same study, the antifoulant tributyltin (TBT), a partial mammalian PPARγ agonist, was shown to be a potent antagonist of the plaice PPARαa.

With the same Gal4 fusion system, but using instead the LBDs of the two Atlantic cod PPARα isoforms for transfections in the mammalian COS-7 cell line, Søderstrøm et al. [38] also confirmed receptor activation by Wy-14643 and demonstrated that this compound is a more potent PPARαb agonist than of PPARαa. Furthermore, the cod PPARαb was shown to be activated by three carboxylated congeners of perfluoroalkyl acids (PFAAs), including PFOA.

In agreement with the results obtained with the plaice receptor, PFOA, or indeed any of the other PFAA congeners, were not able to activate the PPARαa isoform. The authors addressed the discrepancy between the two isoforms by molecular docking analyses and concluded that this effect is due to the more favorable binding position of the agonists in PPARαb, assisted also by the reduced flexibility of the shorter ω-loop of the isoform, as opposed to that of PPARαa. Moreover, they demonstrated that binary combinations of Wy-14643 with PFAAs that exhibit non-agonistic behavior (e.g., the sulfonated congener PFOS) elicited a dose-dependent activation of both PPARα isoforms. Binary combinations of PFOA and PFOS also resulted in the synergistic activation of PPARαb but had no effect on PPARαa activity. Thus, a double-ligand model was proposed, in which the non-agonist molecule occupies a second binding site in the hydrophobic pocket formed by the β-sheet and helices 2 and 3. Accordingly, this leads to the stabilization of the ω-loop and, consequently, to a more stable LBD structure favoring interactions with the coactivator(s). Regarding this double-ligand model, in a later study by the same group, it was observed that while Wy-14643 is a more potent activator of PPARαb, eliciting an isoform response at lower ligand concentrations, the efficiency of PPARαa is three-fold higher at high ligand concentrations [65]. Thus, it would be interesting to investigate whether higher ligand concentrations “force” the entry of a second agonist molecule into the binding cavity of PPARαa, assisted also by the more flexible ω-loop of this isoform. The binding of two Wy-1463 molecules to the human PPARα LBD has been previously demonstrated [66] and results in the stabilization of the domain, and particularly of the AF-2 (H12). Simultaneously, binding of multiple ligands by the PPAR LBDs is also supported by earlier crystallographic studies of the mammalian receptors (see [67] and references therein).

#### 3.4.2. The fPPARδ LBD

**Figure 2:**
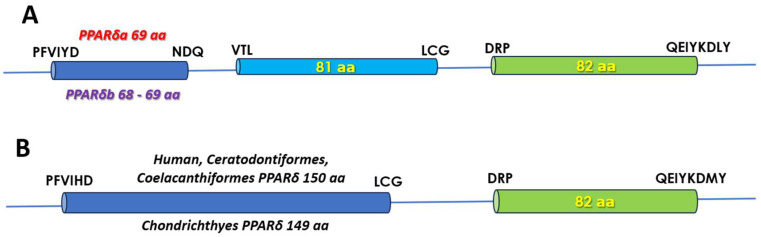
A schematic representation of the exons encoding the LBD of the fPPARδ isoforms. (**A**) The 3-exon Actinopterygian LBD type. (**B**) The 2-exon LBD type common to the mammalian and non-Actinopterygian fish PPARδ. Residues encoded by each exon are indicated above or on the exons. The aa sequences at the exon boundaries are also shown.

In all the fPPARδ genomic sequences examined, the LBD of the receptor is encoded by three exons (Figure 2). In the sequences listed in GenBank as the PPARδa isoform, the encoded domain contains 232 aa residues, i.e., as in the mammalian, lungfish, and coelacanth receptor (Figure 2B). The same number of residues is also encountered in the PPARδb isoform of Salmoniformes, Esociformes, Osmeriformes, and Anguilliformes. However, in the species of the Characiformes, Clupeiformes, Cypriniformes, Siluriformes, and Gymnodontiformes Orders, the domain length is one residue shorter due to a deletion in the first exon, and specifically in the region of the ω-loop (equivalent position of V232 in the human receptor, GenBank NM_006238). Interestingly, the same deletion is observed in the chondrichthyan receptor.

For both isoforms, between-species sequence identity in this domain is high (≥90%), as is also the case between the two isoforms within species. As is the case in fPPARα, the majority of the residues important for ligand binding are conserved in both PPARδ isoforms, with the most important difference being in the Arm I cavity of the binding site where H413 in the human receptor is substituted by Asn in both fish isoforms (Table 1).

For this fPPAR isotype, the direct interaction of ligands/activators with the receptor has only been demonstrated with the European sea bass PPARδb isoform [17], with the receptor shown to efficiently bind fatty acids and ETYA, but not the mammalian PPARα-specific ligand Wy-14643. Through transient transfection experiments with the plaice and sea bream native PPARδb, Leaver et al. [18] confirmed the above results and, further, showed that the receptor does not respond to the mammalian PPARγ-specific ligands rosiglitazone and GW1929. Interestingly, in these experiments, and in contrast to the sea bream receptor, the plaice PPARδb was poorly activated by natural fatty acids. A lack of activation by natural fatty acids was also observed with the pufferfish and the medaka receptor [25,63]. In further transfection experiments with the plaice PPARδb, but using the LBD of the receptor fused to the GAL4 DBD instead of the native protein, Colliar et al. [64] showed a significant activation of the receptor with the palmitoleic, linoleic, stearidonic, and EPA fatty acids, in addition to bromopalmitate, gemfibrozil, mono-1methyl-hexyl-phthalate, and the mammalian PPARδ-specific ligand GW501516. As was the case with the plaice PPARαa, tributyltin was found to inhibit the activity of the receptor but with reduced potency as compared to PPARαa.

In transient transfection experiments with the two Atlantic salmon isoforms, originally described as PPARβ1A and β2A, Leaver et al. [24] showed that PPARδ1A, although poorly activated by natural fatty acids, exhibited a strong response to bromopalmitate and to GW501516. In contrast, the δ2A isoform was not responsive to any of the compounds tested and, in fact, reduced the basal transcription level of the reporter gene. This repressive effect was also demonstrated in co-transfection experiments of both isoforms and in the presence of the PPARδ1A activator GW501516, leading to the hypothesis that the function of PPARδ2A, which is highly expressed in the gills of salmon, may be to prevent the activation of other PPARs by exogenous compounds or contaminants. In addition, the inactivation of the δ2A isoform by PPARδ-specific ligands could not be attributed to residues important for ligand binding, as the LBD in both isoforms is practically identical. Therefore, other structural features of the receptor, and particularly the A/B domain in which the two isoforms diverge significantly, may be responsible for this effect.

Using the cod PPARδb LBD fused to the Gal4 DBD in transfection experiments, Søderstrøm et al. [38] also demonstrated the activation of the receptor by GW501516. As in the case of the cod PPARα isoforms, and according to the double-ligand model proposed by the authors, combined exposure of the receptor’s LBD to GW501516 and PFAA congeners, and especially to nonadecafluorodecanoic acid (PFDA), significantly and synergistically increased its transcriptional activity.

#### 3.4.3. The fPPARγ LBD

**Figure 3:**
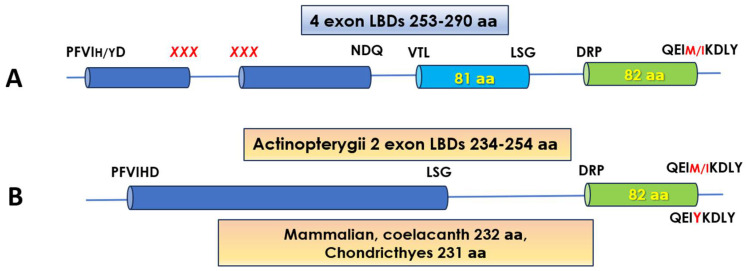
A schematic representation of the exons encoding the LBD of the fPPARγ isoforms. (**A**) The 4-exon LBD of Actinopterygian PPARγ. (**Β**) The 2-exon LBD type common to the mammalian, Actinopterygian, and non-Actinopterygian fish PPARγ. The number of encoded residues is indicated, where applicable, on the exon representation. The aa sequences at the exon boundaries are also shown. The sequence *XXX* in the “4-exon” type (panel **A**) indicates variable residues.

Of the three fish isotypes/isoforms’ LBDs, that of the fPPARγ is the most perplexing both in terms of structure and function. As depicted in Figure 3, the domain, in the majority of species examined and depending on the Order they belong, is encoded by either two or four exons. However, in the species where duplicate genes of the receptor have been identified, in the Salmoniform and Esociform species, the LBD of the isoform termed “γa” is encoded by four exons and the “γb” by two exons, while in the Osmeriform, Characiform, Clupeiform, and Siluriform ones, the domain of both isoforms is encoded by two exons.

The “4-exon type” LBD varies considerably in length due to the ω-loop sequence. The loop spans exons 1 and 2 of the LBD and, consequently, the flanking aa residues at these exon boundaries are not conserved (marked in red as *XXX*, Figure 3A), although a Gln stretch is often observed at the boundary of exon 1. The longest ω-loop insertion (59 aa) was observed in the Atlantic salmon receptor (GenBank: XM_014168482.2) and the shortest (24 aa) in the two Sparidae species examined (*Sparus aurata* and *Acanthopagrus latus*). The primary structure of the loop appears to be conserved between species of different Orders, as the sequence LTAGHGG(I/L/V)TG(A/V)HXGS(E/D)CGVLGM(T/A), with deletions or insertions, is observed in all but the Salmoniform, Esociform, Gadiform, and Lampriform species. This sequence bears no similarity to the ω-loop of fPPARαa. Between species, sequence identity for this domain type is >80%.

The two-exon-encoded “γb” isoform LBD of the Atlantic and chum salmon has 241 aa residues, with the ω-loop extended by 9 aa residues, and of sequence RSVLPPEEP, in both species. The same ω-loop sequence is also observed in the other Salmonoid species in which the “γb” isoform has been identified, i.e., the rainbow trout and the Sunapee trout (*Savelinus alpinus*). In contrast, in the Esociform species, northern pike, the ω-loop of the “γb” isoform is extended by only two residues. Sequence identity of this domain between the salmonoid species exceeds 90% and is at 80% between the Salmonoids and northern pike.

In the Characiform *Astyanax mexicanus* (blind cave fish), in which the LBD of both PPARγ isoforms is encoded by two exons, the “γa” has the ω-loop extended by 13 aa residues and that of “γb” by 4 aa. In the other two Characiform species (*Pygocentrus nattereri* and *Colossoma macropomum*), in which only the PPARγa isoform has thus far been identified, the ω-loop is also extended by 13 aa residues and is related to the *Astyanax* sequence. Characteristic of the loop in these species is the prevalence of charged and polar residues (R, E, and particularly Q). The two isoforms in *Astyanax mexicanus* share ≈60% sequence identity at the LBD.

The 2-exon-encoded PPARγa LBD of the Osmeriform species contains a 22 aa residue insertion in the ω-loop and that of the γb isoform 18 aa. As in the case of the Characiform PPARγa, the ω-loop in Osmeriforms is particularly rich in Gln, but also in Pro residues. The within-species sequence identity between the two isoforms’ LBDs is 65%.

In the Clupeidae, the ω-loop sequence length varies between species in either isoform, from 11 to 14 aa in the “γa” and from 6 to 8 residues in the “γb”. Between species, sequence identity of the entire domain, for either the “γa” or the “γb” isoform, is at 80%. Within species, identity of the two isoforms is at 63% for the sardine and 67% for the herring.

In the Siluriform species, residue insertion at the ω-loop of the PPARγa isoform accounts for the extension of the LBD sequence by 12 aa, relative to the mammalian receptor. In contrast, the loop is extended by only two aa residues in the “γb” isoform. The inserted loop sequence is highly conserved in the species of this Order. Sequence identity within species for the two isoforms is at 56%. Between species, the identity is 97% for PPARγa and 85% for PPARγb.

For the remaining species in Orders in which the PPARγ LBD is encoded by two exons and only one isoform has thus far been identified, the LBD sequence varies in length. Thus, in the Acipenseriformes and Polypteriformes, it contains 235 aa, in the Cypriniformes, 238 aa; in the Elopiformes, Osteoglossiformes, Semionontiformes, and Gonorynchiformes, 241 aa; and in the Gymnotiformes, 243 aa, with either a 3, 6, 9, or 11 aa residue insertion in the ω-loop, respectively. The loop sequence does not present a common motif between these Orders but is highly conserved in all Cypriniformes species examined. The presence of an extended ω-loop in the Acipenseriformes and Polypteriformes Orders suggests that the residue insertion event in this PPAR isotype coincides with the early Actinopterygian evolution.

Phylogenetically, it appears that the LBD of the PPARγa isoform in Osmeriformes, Characiformes, Clupeiformes, and Siluriformes is more closely related to the PPARγ LBD sequence of the Cypriniformes, Elopiformes, Gymnotiformes, Gonorynchiformes, and Osteoglossiformes, (Figure S1). In contrast, a more distant relation is observed between the PPARγb isoform of Osmeriformes, Characiformes, and Siluriformes, as well as with the LBD sequences of the Acipenseriformes and Semionotiformes PPARγ. Despite this discrepancy, for the data presented in Table 1, the Acipenseriformes, and Semionotiformes sequences are considered as homologous to the PPARγb isoform type, while those of the Cypriniformes, Gymnotiformes, Gonorynchiformes, Elopiformes, and Osteoglosiformes, are considered as homologues to PPARγa.

In contrast to the fPPARα and δ isotypes that have been shown to be activated by their respective natural and synthetic mammalian ligands (see above), natural fatty acids as well as the mammalian high-affinity PPARγ-specific ligands (e.g., rosiglitazone, GW1929) fail to activate the fish receptor. This failure has been attributed to residues important for receptor–ligand interactions not being conserved in the fPPARγ [18,46,64,68,69]. Indeed, as shown in Table 1, the fPPARγ isotype contains more substituted residues in the ligand binding site than either the fPPARα or δ isotypes. Most importantly, these substitutions include two out of the four residues in Arm I that form hydrogen bonds with the acidic group of the ligands in the mammalian receptor, i.e., H323 and Y473. In all fish species of the 4-exon LBD PPARγa type, as well as in most of the 2-exon “γa” type examined, the equivalent position of H323 is occupied by Ile (Met in the European sardine). In the PPARγb isoform of Salmoniformes, Esociformes, Characiformes, Elopiformes, and Osteoglossiformes, Ile is also encountered at this position, while a Met residue is present in the Clupeidae and Osmeridae species. In contrast, the receptor from Acipenseriformes, Semionotiformes, and Polypteriformes, which we have arbitrarily assigned to the PPARγb isoform, retains the His residue. Most importantly, in all Actinopterygii, the Y473 is replaced by Met and in rare cases by either Leu or Ile. Being located in helix 12, Y473, through hydrogen bonding with the ligand, holds the AF-2 in an active confirmation for co-activator recruitment (see [58] and ref. therein), an effect that cannot be produced by the non-polar Met, Ile, or Leu residues. Interestingly, Y473 is conserved in the Chondrichthyan species, as well as in the coelacanth but not in the lungfish *Protopterus annectens* (XM_044078056.1) (Figure 3, Table S2). In fact, most of the ligand binding residues are conserved in the Chondrichtyan and coelacanth receptor (Table S2). Indeed, Capitão et al. [69] demonstrated that the mammalian PPARγ-specific ligand rosiglitazone can activate transcription through the LBD of little skate (*Leucoraja erinacea*). Furthermore, it was shown by both transient transfection and ligand binding assays that the little skate PPARγ can bind to the partial human PPARγ agonist tributyltin (TBT). In previous studies with plaice PPARγ, TBT, like all other natural or synthetic compounds tested, was not effective in transactivation experiments and it was indeed shown to act as a repressor of plaice PPARαa and δa [64]. A lack of activation by this compound was also observed with the zebrafish (*Danio rerio*) PPARγ [69].

In a study aimed to assess interspecies differences in response to endocrine disrupting chemicals, Garoche et al. [21] also used the zebrafish PPARγ, along with the human and *Xenopus* receptor. Interestingly, and in contrast to previous studies, the zebrafish receptor was shown to be responsive to several of the compounds tested, albeit with various efficiencies. In particular, two organophosphorus compounds, triphenyl phosphate (TPP) and tri-*o*-tolyl phosphate (ToTP), exhibited strong agonistic behavior, which was also specific to the fish receptor. Furthermore, the human LXR ligand, GW3965, also specifically and strongly activated the zebrafish PPARγ. Molecular modeling studies located the binding of the above compounds in the hydrophobic sub-pocket of the LBD formed by helices H3 and H5 and the β-sheet, in both the human and the zebrafish receptor. This binding does not allow contacts of the compound(s) with the AF-2 (H12), i.e., contacts that determine the agonistic behavior of the mammalian PPARγ ligands and, particularly, the hydrogen bond with Y473 of the receptor. However, as the equivalent Y473 position is substituted by Met in the zebrafish PPARγ, the AF-2 involvement in the activation of the zebrafish receptor appears not to be a prerequisite.

Support for the AF-2-independent activation of the fPPARγ comes from a study that demonstrated the activation of both European sardine PPARγ isoforms by TBT [40]. TBT also binds to the hydrophobic sub-pocket in the human receptor and stabilizes the LBD by bond formation between the tin atom and C285 [70]. In a previous study by the same group [69], it was reported that TBT could not activate the zebrafish PPARγ, an effect that was attributed to the substitution of C285 by Tyr. However, C285 is also substituted in the sardine receptor by an aromatic residue (Phe). The discrepancy between the two fish receptors was explained by the presence of a Met residue in the S3 β-strand of the β-sheet, only in the sardine receptor, which has the potential of bond formation with the tin moiety of TBT, albeit with lower efficacy compared to Cys. However, it should be noted that the C285 substitution in the PPARγ receptor appears to be a very rare event in the teleost fish lineages. From all fPPARγa sequences examined, this substitution was observed only in the two Danionidae species, *D. rerio* and *D. aesculapii* (Cypriniformes), and the European sardine. Consequently, if this residue is critical for receptor activation by TBT, it would be expected that all fish receptors that maintain Cys at that position would be responsive to the compound. However, as noted above, this was not the case with the plaice PPARγ. Therefore, the activation of the sardine’s receptor by TBT may involve other unique structural features in the LBD of this species.

## 4. The Expression of the PPAR Isoforms in Fish Tissues

Fish represent the most diverse and largest group of all vertebrates that includes marine, anadromous, and fresh-water species, inhabiting environments as diverse as abyssal depths (*Pseudoliparis belyaevi* < 8000 m) and high-altitude lakes and streams (Tibetan loach, *Triplophysa stolickai*, >5000 m). Fish, being also poikilotherms, have adapted their energy homeostasis mechanisms to the environmental conditions of their habitat range and, consequently, to the dietary energy sources available. Lipids serve as the principal energy source in fish, with species utilizing different tissues to store it (liver, muscle, peritoneal cavity). Consequently, it is expected that transcription factors, as PPARs, which are involved in the regulation of lipid metabolism, will not present the same tissue expression pattern in all fish species. Indeed, this is supported by the presently available data, as in only a handful of species, a similar, but not identical, pattern of expression has been observed. Important to note is that the majority of the published reports concern the expression pattern of only one of the receptors’ isoforms. As the differential expression of the PPAR isoforms can provide clues on their biological functions, the discussion here is limited to the few fish species in which the tissue expression profile of both isoforms of the isotype(s) has been examined in the same sampled specimens.

The expression of both PPARα isoforms has been studied in the turbot (*Scopthalmus maximus*) [32], the Japanese sea bass (*Lateolabrax japonicus*) [35], and the loach (*Misgurnus anguillicaudatus*) [34]. In general, the two isoforms exhibited a species-specific tissue distribution pattern. Thus, in the turbot, PPARαa was most abundantly expressed in the heart with lower expression in the kidney, gill, liver, brain, and muscle. PPARαb was also highly expressed in the heart as well as in the kidney but, in terms of transcript abundance, its expression in the heart was two-fold lower as compared to that of PPARαa. In the Japanese sea bass, PPARαa was ubiquitously expressed in the tissues tested and most abundant in the stomach and adipose tissue but with low expression in the liver. In contrast, PPARαb had a more restricted tissue distribution and was most highly expressed in the liver and moderately in the adipose tissue. Similarly, in the loach, both isoforms were detected in all tissues tested, exhibiting a somewhat different profile as PPARαa was predominantly expressed in the heart and PPARαb in the liver. In terms of absolute expression levels, the PPARαb copy numbers were, in all tissues, two-fold higher than those of PPARαa.

The expression of the PPARδ isoforms was also studied in the loach [39]. Both isoforms were found to have a wide tissue distribution, with PPARδa being most abundantly expressed in the liver and the gonads of both sexes. PPARδb was also strongly expressed in the gonads but compared to PPARδa, its expression was 10- to 100-fold lower in all the other tissues. Transcripts of this isoform were not detected in the kidney or spleen.

The expression of the two PPARδ genes of Atlantic salmon, originally described as PPARβ1A and PPARβ2A [24], differed essentially only in the liver and gill, with the 1A isoform being predominantly expressed in the liver and the 2A in the gills, while comparable levels of the two isoforms’ transcripts were detected in the other tissues tested [24].

The only species in which the tissue expression of both PPARγ isoforms has thus far been studied is that of the European sardine [40]. PPARγa was found to be ubiquitously expressed in the tissues examined with its transcripts being most abundant in the brain, midgut, gill, and intestine. PPARγb was also highly expressed in the midgut, albeit at lower levels than PPARγa, and moderately expressed in the liver and kidney and at low levels in the brain. The isoform was not detected in the gills, gonads, and muscle.

Although not concerning a PPARγ isoform, it is worth noting the diverse tissue- and developmental-stage-dependent pattern of expression of the two transcripts, which result from alternative promoter usage in the PPARγ gene of the yellow catfish (*Trachysurus fulvidraco*) [46].

The expression pattern of both the PPARα and δ isoforms was also studied in the early developmental stages of the loach [34,39]. Transcripts of both PPARα and PPARδ isoforms were detected in spermatozoa and the unfertilized egg, and in most of the developmental stages examined. A different expression pattern between the PPARα isoforms was observed after the blastula stage, while the PPARδ isoforms exhibited similar expression throughout development. The expression of both PPAR isotypes in the gonads and in the early developmental stages suggested that the fish receptors, like their mammalian counterparts, are involved in germ cell energy metabolism, oocyte maturation, and organ development [39].

The differential tissue expression of the PPARα and PPARδ isoforms has also been demonstrated in several species and in response to diets containing different fatty acid profiles [34,35,37,39], as well as in response to heat stress [63,71,72], underscoring the involvement of the PPAR isotypes/isoforms in the regulation of metabolism and energy balance in fish species.

According to the above limited data, the expression of the PPAR isoforms, although presenting a differential pattern under normal or challenged conditions, still shows a significant tissue overlap. Therefore, the expression of either isoform does not seem to act as a “turn-off switch” for the other. This may imply that the isoforms are involved in the regulation of different biochemical pathways, possibly responding to different ligands/activators and/or interacting differently with the other components of the transcriptional machinery on the promoters of target genes. Alternatively, isoform-specific structural features may confer differential stability properties to the receptors, thus securing the uninterrupted regulation of target genes under conditions that render one of the isoforms less stable. Addressing these questions is crucial to understanding the functions of the duplicate PPAR genes and the advantages they may present in the physiology of fish, regarding their adaptation to different environments and to their nutritional requirements.

## 5. Conclusions

The study of fPPARs spans more than two decades, a period during which the results produced have significantly increased our understanding of the role of these receptors in fish physiology and biochemistry. Nevertheless, there is still a number of questions that need to be addressed in order to precisely determine their functions in the biochemical pathways in which they are involved. Presently, there is only indirect evidence for genes that are targets of fPPAR action, deduced either from the known involvement of the mammalian receptors in their regulation, or from the concomitant, with that of the receptor(s), up- or down-regulation of their expression, in response to chemical or physical stimuli that are expected to activate PPAR-driven transcription. Potential PPREs have been identified in the promoters of a few fish genes (*fabp*, *fads2*, *gsta1*), and these promoters could be used to decipher the interactions of fPPARs with the element itself, as well as with the other components of the transcriptional complex. Furthermore, studies on the molecular mechanisms/transcription factors controlling the expression of the fPPAR genes are presently lacking. Regarding the above, it is interesting that the interactions of the fish receptors with co-activators have not as yet been examined, despite the fact that the PPARγ coactivator-1α (PGC-1α) has been identified in several fish species.

As for the structural features of the receptors, two regions present particular interest for further studies, i.e., the A/B domain and the ω-loop of the LBD. Concerning the latter, its impressive variability in terms of sequence length and residue composition suggests potentially important functional differences between species, at both the ligand recognition and binding affinity levels. In addition, the Actinopterygian/Teleost-specific ω-loop sequence expansion appears to be associated with the divergence of the different lineages, while in some of the fPPARγ genes, it is directly linked to the insertion of an extra intron in the gene structure.

The most mysterious of the fish receptors is PPARγ, for which no strong natural agonist has been identified. Although this receptor may not require the involvement of AF-2 for transactivation, it still remains to be seen whether a lack of response to the mammalian PPARγ-specific ligands results solely from the substitution of Y473 in the AF-2, or whether other factors are also involved.

## Figures and Tables

**Table 1:** Residues defining the ligand binding cavity of the PPAR isotypes (adapted from [58]). hPPAR: human PPAR. Residue numbering is according to GenBank sequences XM_011530240.1, NM_006238.5, and NM_138712.5 for the hPPARα, δ, and γ isotypes, respectively. C.C. refers to the “Charge-Clamp” residues involved in interactions with co-activators. Residues in parenthesis are more rarely encountered in the fish sequences examined (at least two species of each Actinopterygian Order of Table S1, when applicable). Shaded residues indicate differences between the human and fish receptors. Arm I residues in bold are important for hydrogen bond formation with the polar head of ligands. In the fPPARγ isotype, X stands for an exon in the 4- and 2-exon type LBD.

	hPPARα	fPPARαa	fPPARαb	hPPARδ	fPPARδa	fPPARδb	hPPARγ	fPPARγa_4x	fPPARγa_2x	fPPARγb
Arm I	F273	F	F	F245	F	F	F282	F	F	F
	C276	C	C	C249	C	C	C285	C	C(F/W)	V/C(I/Y)
	Q277	Q	Q	Q250	Q	Q	Q286	Q	Q	Q
	**S280**	S	S	**T253**	T	T	**S289**	S	S	S(L/Q)
	**Y314**	Y	Y(H)	**H287**	H	H	**H323**	I	I(M)	I(M/H/F)
	I317	L	L(M)	I290	I	I	I326	L	L	L/M(N)
	F318	F	F	F291	F	F	Y327	I(T)	I(F/S)	I(T/M)
	I354	M	M	I327	I	I	F363	M(I)	M(I/L)	M(I)
	**H440**	H	H	**H413**	N	N	**H449**	H	H	H
	V444	V(I)	V(I)	M417	V	V	L453	I(M)	Q(E)	I
	L460	L	L	L433	L	L	L469	L	L(M)	L
	**Y464**	Y	Y	**Y437**	Y	Y	**Y473**	M(I/L)	M(I)	M(I)
Arm II	I241	I	I	I213	I	I(V)	I249	I	I(V)	I(V)
	L247	L	L(F)	L219	L	L	L255	L	L	L(M)
	E251	E	E(Q/R)	E223	E	E	E259	E(Q)	E(Q)	Q(E)
	L254	L	L(F)	W228	W	W	F264	C	C(F/S)	C(F/S/R)
	I272	L	I(L)	V245	V	V	I281	F(L)	F(L)	F(M/V/I)
	C275	C	C	R248	R	R	G284	S	S(R)	R(Q/S)
	M330	L	L	L303	L	L	V339	T	T	T(V/M/A)
	V332	V	V	V341	V	V	I341	I(M)	I(V)	F/M(L/I/C)
	I339	I	I	V348	V	V	M348	M	M	M(I)
	F343	F	F	F305	F	F	F352	F	F	F
	L344	L	L	L317	L	L	L353	L(I)	L	L(I/F)
	M355	M	M	I328	M	M	M364	M(L)	M	M(L/I)
Entrance	M220	M	M	M192	M	M	L228	L	L	L(I/T)
	T279	T(I/A/F)	T	T252	T(S)	T	R288	R	R	R(G/C)
	E282	E	E	E255	E	E	E291	E	E	E
	T283	T	T	T256	T	T	A292	A	A	A(S/T/C/G)
	E286	E	E(Q)	E259	E(Q)	E(Q)	E295	E	E	E(R/D/Q)
	L321	L	L	L294	L	L	L330	M(W)	M	L(W/M/V/T)
	V324	C	S/C	I297	L	I(L)	L333	L	L	L(F)
	A333	A	A	A306	A	A	S342	S(A)	S(A)	A/S(G)
	Y334	R(S)	Y(H/Q/R/S)	N307	N	N	E343	Y	Y	Y(E/S/C/D)
C. C.	K292	K	K	K265	K	K	K301	K	K	K
	E462	E	E	E435	E	E	E471	E	E	E

## Supplementary Materials

The following supporting information can be downloaded at: https://www.mdpi.com/article/10.3390/biom14060634/s1, Table S1: GenBank Accession numbers of Actinopterygian PPAR isotypes/isoforms; Table S2: Residues defining the ligand binding cavity of huma, lungfish coelacanth and chondrichthyan PPARγ; Figure S1: Phylogenetic tree (Maximum Likelihood) for the LBDs of the PPARγ isoforms from different fish Orders.
