# Supplementary material for: The Peroxisome Proliferator-Activated Receptors of Ray-Finned Fish: Unique Structures, Elusive Functions
Source: Biomolecules. 2024 May 29;14(6):634. doi: 10.3390/biom14060634 (PMC11201486; doi:10.3390/biom14060634)
Supplement: Supplementary file 1 [file biomolecules-14-00634-s001.zip › Figure S1.pdf]

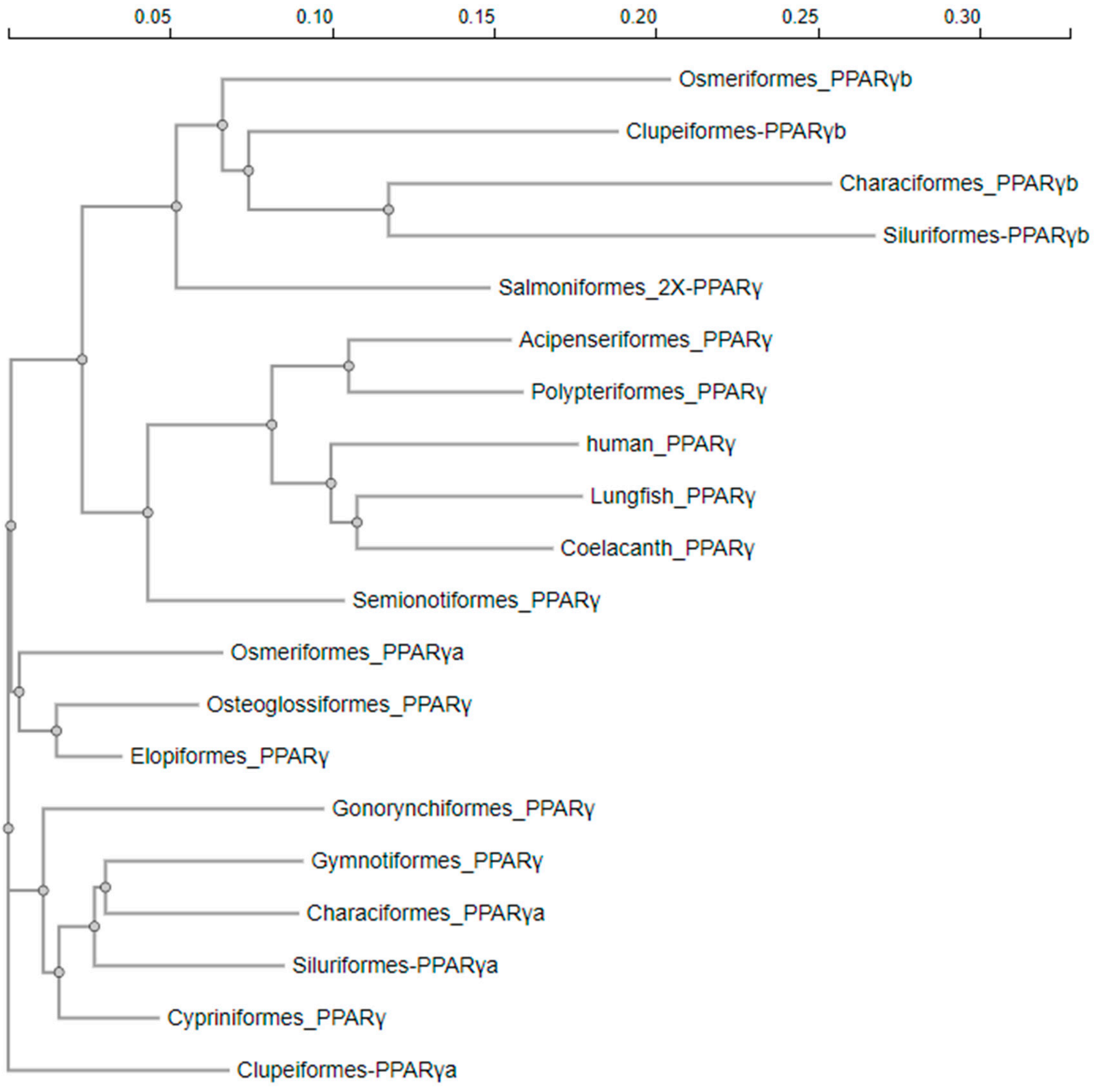

Figure S1. Phylogenetic tree (Maximum Likelihood) for the LBDs of the PPARγ isoforms from different fish Orders.
