# Supplementary material for: The Peroxisome Proliferator-Activated Receptors of Ray-Finned Fish: Unique Structures, Elusive Functions
Source: Biomolecules. 2024 May 29;14(6):634. doi: 10.3390/biom14060634 (PMC11201486; doi:10.3390/biom14060634)
Supplement: Supplementary file 1 [file biomolecules-14-00634-s001.zip › Table S1.pdf]

**Table S1.** GenBank Accession numbers of Actinopterygian PPAR isotypes/isoforms

**PPARα sequences**

| ORDER | Species | Isotype | GenBank Access. no |
|---|---|---|---|
| Acanthuriformes | Siganus canaliculatus | PPARαa | JF502070.1 |
| | Scatophagus argus | PPARαa | XM_046378703.1 |
| | | PPARαb | XM_046393672.1 |
| Acipenseriformes | Acipenser gueldenstaedtii | PPARαb | KR906071.1 |
| | Polyodon spathula | PPARαb | XM_041255284 |
| Acropomatiformes | Lateolabrax japonicus | PPARαa | KM502870.1 |
| | | PPARαb | FJ208989.1 |
| Anguilliformes | Anguilla anguilla | PPARαb | XM_035427651 |
| | Congridae; Conger | PPARαa | XM_061230003 |
| Anabantiformes | Anabas testudineus | PPARαb | XM_026365231 |
| | Betta splendens | PPARαa | XM_029160787 |
| | | PPARαb | XM_029153261.2 |
| Atheriniformes | Melanotaenia boesemani | PPARαa | XM_041976917 |
| | | PPARαb | XM_041997151 |
| Batrachoidiformes | Thalassophryne amazonica | PPARαa | XM_034162915.1 |
| | | PPARαb | XM_034176034.1 |
| Beloniformes | Oryzias sinensis | PPARαa | KP271475 |
| | Oryzias melastigma | PPARαa | XM_024282897 |
| | | PPARαb | XM_024281160 |
| | Oryzias latipes | PPARαa | NM_001164875.1 |
| | Cololabis saira | PPARαa | XM_061714954.1 |
| | Cololabis saira | PPARαb | XM_061721110.1 |
| Blenniiformes | Gouania willdenowi | PPARαa | XM_028449304 |
| Carangaria incertae sedis | Centropomus viridis | PPARαa | MH397707 |
| | Lates calcarifer | PPARαa | XM_018687523.2 |
| | | PPARαb | XM_018675942.2 |
| | Toxotes jaculatrix | PPARαa | XM_041029906.1 |
| | Toxotes jaculatrix | PPARαb | XM_041037668.1 |
| Carangiformes | Seriola lalandi dorsalis | PPARαa | XM_023402033.1 |
| | Seriola dumerili | PPARαa | XM_022749385.1 |
| | Seriola aureovittata | PPARαb | XM_056387397 |
| | Trachinotus ovatus | PPARαa | KP893147 |
| | Rachycentron canadum | PPARαa | EF680883.1 |
| | Echeneis naucrates | PPARαa | XM_029495069.1 |
| Chaetodontiformes | Chelmon rostratus | PPARαa | XM_041963837 |
| | | PPARαb | XM_041939607 |
| Characiformes | Astyanax mexicanus | PPARαa | XM_007232266.4 |
| | | PPARαb | XM_007231582.4 |
| | Pygocentrus nattereri | PPARαa | XM_017716832.2 |
| | | PPARαb | XM_017701190.2 |
| | Colossoma macropomum | PPARαa | XM_036577510.1 |
| | | PPARαb | XM_036576389.1 |
| Cichliformes | Pundamilia nyererei | PPARαa | XM_005725145 |
| | Maylandia zebra | PPARαa | XM_004560806.5 |
| | Haplochromis burtoni | PPARαa | XM_005915662.3 |
| | | PPARαb | XM_005927826.3 |
| | Astatotilapia calliptera | PPARαa | XM_026146670.1 |
| | Archocentrus centrarchus | PPARαa | XM_030719684.1 |
| | Simochromis diagramma | PPARαa | XM_040026211.1 |
| | Oreochromis niloticus | PPARαa | NM_001290066 |
| | | PPARαb | XM_003443920.5 |
| | Oreochromis aureus | PPARαb | XM_031727356.2 |
| | Neolamprologus brichardi | PPARαb | XM_006782487.2 |

**PPARα cont'd**

| | | | |
|---|---|---|---|
| Clupeiformes | Alosa alosa | PPARαa | XM_048267695.1 |
| | | PPARαb | XM_048256333 |
| | | PPARαb | XM_048256333 |
| | Alosa sapidissima | PPARαa | XM_042078001.1 |
| | | PPARαb | XM_042109303.1 |
| | Clupea harengus | PPARαa | XM_042706932.1 |
| | | PPARαb | XM_012842487.3 |
| Cypriniformes | Carassius gibelio | PPARαa | XM_052577736 |
| | | PPARαb | XM_052597479 |
| | Carassius carassius | PPARαa | XM_059505298.1 |
| | | PPARαb | XM_059536098.1 |
| | Sinocyclocheilus anshuiensis | PPARαa | XM_016495997.1 |
| | Cyprinus carpio | PPARαa | XM_042722106.1 |
| | | PPARαb | XM_042752572.1 |
| | Puntigrus tetrazona | PPARαa | XM_043236699.1 |
| | | PPARαb | XM_043228342.1 |
| | Labeo rohita | PPARαa | XM_051108330.1 |
| | | PPARαb | XM_051100086.1 |
| | Onychostoma macrolepis | PPARαa | XM_058773455.1 |
| | | PPARαb | XM_058767321.1 |
| | Danio rerio | PPARαa | NM_001161333.1 |
| | | PPARαb | NM_001102567.1 |
| | Danio aesculapii | PPARαa | XM_056455645.1 |
| | | PPARαb | XM_056451616.1 |
| | Xyrauchen texanus | PPARαb | XM_052119599 |
| | Myxocyprinus asiaticus | PPARαb | XM_051689998 |
| | Megalobrama amblycephala | PPARαa | XM_048154865.1 |
| | | PPARαb | XM_048158020.1 |
| | Ctenopharyngodon idella | PPARαa | XM_051892469.1 |
| | | PPARαb | XM_051883407.1 |
| | Mylopharyngodon piceus | PPARαa | MW088564 |
| | | PPARαb | MW088565 |
| | Rhinichthys klamathensis goyatoka | PPARαa | XM_056237241.1 |
| Cyprinodontiformes | Kryptolebias marmoratus | PPARαa | XM_017424858.3 |
| | | PPARαb | XM_017434249.3 |
| | Nematolebias whitei | PPARαa | XM_037695712 |
| | | PPARαb | XM_037683651 |
| | Austrofundulus limnaeus | PPARαa | XM_054740057 |
| | Nothobranchius furzeri | PPARαa | XM_054740057 |
| | | PPARαb | XM_054733590.1 |
| | Nothobranchius kadleci | PPARαa | HAEA01012422 |
| | Nothobranchius rachovii | PPARαa | HAEH01020359 |
| | Nothobranchius pienaari | PPARαa | HAEF01004545 TSA |
| | Nothobranchius kuhntae | PPARαa | HAEE01016975 |
| | Aphyosemion striatum | PPARαa | HADW01005939 |
| | | PPARαb | HADW01020240 |
| | Xiphophorus couchianus | PPARαa | XM_028044166.1 |
| | Xiphophorus maculatus | PPARαa | XM_014476054.2 |
| | Xiphophorus hellerii | PPARαa | XM_032589411.1 |
| | Poeciliopsis prolifica | PPARαa | XM_055055787.1 |
| | | PPARαb | XM_055037064.1 |
| | Poecilia mexicana | PPARαa | XM_014985020 |
| | Poecilia formosa | PPARαa | XM_007578224.2 |
| | Gambusia affinis | PPARαb | XM_044124896 |
| | Fundulus heteroclitus | PPARαa | XM_012873529 |
| | | PPARαb | XM_012880334 |
| | Cyprinodon tularosa | PPARαb | XM_038274285 |
| | Girardinichthys multiradiatus | PPARαb | XM_047387995 |

**PPARα cont'd**

| | | | |
|---|---|---|---|
| Esociformes | Esox lucius | PPARαa | XM_010887147.4 |
| | | PPARαb | XM_034288333.1 |
| Eupercaria incertae sedis | Dicentrarchus labrax | PPARαa | AY590300.1 |
| | | PPARαb | XM_051381902.1 |
| | Morone saxatilis | PPARαa | XM_035675293.1 |
| | Larimichthys crocea | PPARαa | XM_010749024.3 |
| Gadiformes | Gadus chalcogrammus | PPARαa | XM_056587966.1 |
| | | PPARαb | XM_056599520.1 |
| | Gadus macrocephalus | PPARαa | XM_060050634.1 |
| | | PPARαb | XM_060060368.1 |
| | Gadus morhua | PPARαa | MW690583 |
| | | PPARαb | MW690582 |
| Gobiiformes | Periophthalmus magnuspinnatus | PPARαa | XM_055225597 |
| | | PPARαb | XM_033968228 |
| | Boleophthalmus pectinirostris | PPARαa | XM_020930096 |
| | | PPARαb | XM_020935338 |
| | Synechogobius ommaturus | PPARαb | JQ707899 |
| Gonorynchiformes | Chanos chanos | PPARαa | XM_030767332.1 |
| Gymnotiformes | Electrophorus electricus | PPARαa | XM_027032075.2 |
| | | PPARαb | XM_027019959.2 |
| Holocentriformes | Myripristis murdjan | PPARαa | XM_030045963.1 |
| Istiophoriformes | Xiphias gladius | PPARαa | XM_040142591.1 |
| | | PPARαb | XM_040132366.1 |
| Labriformes | Labrus mixtus | PPARαa | XM_061030234.1 |
| | | PPARαb | XM_061035890.1 |
| | Labrus bergylta | PPARαa | XM_020639525.2 |
| | Notolabrus celidotus | PPARαa | XM_034686517 |
| | Cheilinus undulatus | PPARαb | XM_041795011.1 |
| Lampriformes | Lampris incognitus | PPARαa | XM_056277677.1 |
| | | PPARαb | XM_056282215.1 |
| Mugiliformes | Mugil cephalus | PPARαa | XM_047574652.1 |
| | | PPARαb | XM_047596441.1 |
| | Liza haematocheila | PPARαa | KJ848472.1 |
| | Chelon labrosus | PPARαa | AY618315.2 |
| | Mugil incilis | PPARαb | MK183038 |
| Osmeriformes | Hypomesus transpacificus | PPARαb | XM_047034682 |
| Osteoglossiformes | Brienomyrus brachyistius | PPARαb | XM_048987514 |
| Ovalentaria incertae sedis | Amphiprion ocellaris | PPARαa | XM_023277231.3 |
| | | PPARαb | XM_023284214.3 |
| | Acanthochromis polyacanthus | PPARαb | XM_022206528.2 |
| Perciformes | Centropristis striata | PPARαa | XM_059326158.1 |
| | | PPARαb | XM_059334586.1 |
| | Epinephelus moara | PPARαa | XM_050035689.1 |
| | | PPARαb | XM_050073646.1 |
| | Epinephelus lanceolatus | PPARαb | XM_033635277 |
| | | PPARαb | XM_049574358 |
| | Plectropomus leopardus | PPARαb | XM_042495547 |
| | Sander lucioperca | PPARαa | XM_031284793.2 |
| | | PPARαb | XM_031282688.2 |
| | Perca fluviatilis | PPARαa | XM_039791719.1 |
| | | PPARαb | XM_039807847.1 |
| | Etheostoma cragini | PPARαa | XM_034863008.1 |
| | | PPARαb | XM_034878113.1 |
| | Gasterosteus aculeatus aculeatus | PPARαb | XM_040162610.1 |
| | Pungitius pungitius | PPARαb | XM_037450594.1 |
| | Gymnodraco acuticeps | PPARαa | XM_034234342.1 |
| | | PPARαb | XM_034218677.1 |
| | Trematomus bernacchii | PPARαa | XM_034131440.1 |
| | | PPARαb | XM_034149451.1 |
| | Sebastes umbrosus | PPARαa | XM_037761084.1 |
| | | PPARαb | XM_037768683.1 |
| | Anoplopoma fimbria | PPARαa | XM_054624989.1 |
| | | PPARαb | XM_054620807.1 |
| | Stegastes partitus | PPARαa | XM_008294864 |
| | Cyclopterus lumpus | PPARαb | XM_034534659 |
| | Pseudochaenichthys georgianus | PPARαb | XM_034084838 |
| | Pseudoliparis swirei | PPARαb | XM_056416506 |

**PPARα cont'd**

| | | | |
|---|---|---|---|
| Pleuronectiformes | Hippoglossus stenolepis | PPARαa | XM_035147111.2 |
| | | PPARαb | XM_035156478.2 |
| | Hippoglossus hippoglossus | PPARαb | XM_034587470 |
| | Platichthys flesus | PPARαb | XM_062389955 |
| | Pleuronectes platessa | PPARαa | XM_053414619.1 |
| | | | |
| | | PPARαb | XM_053427283.1 |
| | Solea senegalensis | PPARαa | JX424080 |
| | | PPARαb | XM_044036374.1 |
| | Solea solea | PPARαb | XM_058645182 |
| | Cynoglossus semilaevis | PPARαa | XM_008315872.3 |
| | Scophthalmus maximus | PPARαa | XM_047336020.1 |
| | | PPARαb | XM_035641788.2 |
| Salmoniformes | Oncorhynchus mykiss | PPARα1 | XM_021617918.2 |
| | | PPARα2 | XM_021598925.2 |
| | Oncorhynchus kisutch | PPARa2 | XM_020456442 |
| | Oncorhynchus tshawytscha | PPARα2 | XM_024378757 |
| | Coregonus clupeaformis | PPARα1 | XM_041837130 |
| | Salmo salar | PPARα2 | XM_045698635.1 |
| | | PPARα1 | XM_014169857 |
| | Salvelinus alpinus | PPARα2 | XM_023985138 |
| Scombriformes | Thunnus albacares | PPARαa | XM_044185657.1 |
| | | PPARαb | XM_044355484 |
| | Thunnus maccoyii | PPARαb | XM_042411797 |
| | Scomber japonicus | PPARαb | XM_053318575 |
| | Scomber scombrus | PPARαb | XM_062421459 |
| Siluriformes | Ictalurus punctatus | PPARαa | XM_017494484.3 |
| | | PPARαb | XM_017485720.3 |
| | Ictalurus furcatus | PPARαa | XM_053649755.1 |
| | | PPARαb | XM_053641213.1 |
| | Pangasianodon hypophthalmus | PPARαa | XM_034313315.2 |
| | | PPARαb | XM_053236651.1 |
| | Tachysurus fulvidraco | PPARαa | XM_027151209.2 |
| | | PPARαb | XM_027140852.2 |
| | Tachysurus vachellii | PPARαb | XM_060887466 |
| | Silurus meridionalis | PPARαa | XM_046873100.1 |
| | | PPARαb | XM_046859047.1 |
| | Clarias gariepinus | PPARαa | XM_053508188.1 |
| | | PPARαb | XM_053475784.1 |
| | Hemibagrus wyckioides | PPARαa | XM_058416743.1 |
| | | PPARαb | XM_058408305.1 |
| | Neoarius graeffei | PPARαa | XM_060903217.1 |
| | | PPARαb | XM_060914567.1 |
| Spariformes | Acanthopagrus latus | PPARαb | XM_037105928 |
| | Acanthopagrus schlegelii | PPARαb | KX066234 |
| | Dentex dentex | PPARαa | EF470300 |
| | Pagrus major | PPARαb | AB298547.1 |
| | Sparus aurata | PPARαa | AY590299.1 |
| | | PPARαb | DQ232872.1 |
| Syngnathiformes | Doryrhamphus excisus | PPARαb | XM_058074686 |
| | Corythoichthys intestinalis | PPARαb | XM_057836786 |
| | Entelurus aequoreus | PPARαb | XM_062061239 |
| | Nerophis ophidion | PPARαb | XM_061916864 |
| | Syngnathoides biaculeatus | PPARαb | XM_061822527 |
| | Phyllopteryx taeniolatus | PPARαb | XM_061773563 |
| | Hippocampus zosterae | PPARαa | XM_052055203.1 |
| | | PPARαb | XM_052060778.1 |
| | Synchiropus splendidus | PPARαb | XM_053884776.1 |
| | Dunckerocampus dactyliophorus | PPARαa | XM_054753451.1 |
| | | PPARαb | XM_054777788.1 |
| | Phycodurus eques | PPARαa | XM_061668298.1 |
| | | PPARαb | XM_061677962.1 |
| | Syngnathus acus | PPARαb | XM_037254749.1 |
| | Syngnathus typhle | PPARαb | XM_061283581 |
| | Syngnathus scovelli | PPARαa | XM_049760886 |
| | | PPARαb | XM_049724427.1 |
| | Synchiropus splendidus | PPARαa | XM_053861587.1 |
| Tetraodontiformes | Takifugu flavidus | PPARαa | XM_057021790 |
| | | PPARαb | XM_057052850 |
| | Takifugu rubripes | PPARαb | NM_001097630 |

## PPARδ sequences

| ORDER | Species | Isotype | GenBank Access. no |
|---|---|---|---|
| Acipenseriformes | Acipenser ruthenus | PPARd- like | XM_034052266.3 |
| | | | XM_034908036 |
| Anabantiformes | Scatophagus argus | PPARdb | XM_046395905 |
| | Betta splendens | PPARdb | XM_029156008 |
| | Anabas testudineus | PPARdb | XM_026367907 |
| Anguilliformes | Anguilla anguilla | PPARdb | XM_035382859 |
| | | PPARd- like | XM_035387271 |
| | Conger conger | PPARdb | XM_061257343 |
| Atherinoformes | Melanotaenia boesemani | PPARdb | XM_042004905 |
| Batrachoidiformes | Thalassophryne amazonica | PPARdb | XM_034172316 |
| Beloniformes | Oryzias latipes | PPARdb | NM_001278907 |
| | Oryzias melastigma | PPARdb | XM_024293540 |
| | Cololabis saira | PPARdb | XM_061736249 |
| Blenniiformes | Gouania willdenowi | PPARd | XM_028452504.1 |
| | Salarias fasciatus | PPARd | XM_030118413 |
| Carangaria incertae sedis | Lates calcarifer | PPARdb | XM_018665978 |
| | Toxotes jaculatrix | PPARdb | XM_041064851 |
| Carangiformes | Seriola dumerili | PPARdb | XM_022741939 |
| | Seriola lalandi dorsalis | PPARd | XM_023427623 |
| | Seriola aureovittata | PPARdb | XM_056385055 |
| | Echeneis naucrates | PPARd | XM_029506954 |
| Centrarchiformes | Siniperca chuatsi | PPARdb | XM_044212360 |
| | Micropterus dolomieu | PPARdb | XM_046043714 |
| | Micropterus salmoides | PPARdb | XM_038722719 |
| Chaetodontiformes | Chelmon rostratus | PPARdb | XM_041946278 |
| Characiformes | Astyanax mexicanus | PPARda | XM_022682487 |
| | | PPARdb | XM_022671063 |
| | Pygocentrus nattereri | PPARda | XM_037532291 |
| | | PPARdb | XM_017722410 |
| | Colossoma macropomum | PPARda | XM_036559453 |
| | | PPARdb | XM_036578404 |

**PPARδ cont'd**

| | | | |
|---|---|---|---|
| Cichliformes | Oreochromis niloticus | PPARdb | NM_001289636 |
| | Maylandia zebra | PPARdb | XM_004558684 |
| | Haplochromis burtoni | PPARdb | XM_005914849 |
| | Neolamprologus brichardi | PPARdb | XM_006785588 |
| | Astatotilapia calliptera | PPARd | XM_026153966 |
| | Archocentrus centrarchus | PPARd | XM_030732948 |
| | Oreochromis aureus | PPARdb | XM_031746955 |
| | Simochromis diagramma | PPARdb | XM_040047603 |
| Clupeiformes | Clupea harengus | PPARda | XM_031567306 |
| | | PPARdb | XM_012820125 |
| | Alosa sapidissima | PPARda | XM_042088872 |
| | | PPARdb | XM_042097868 |
| | Alosa alosa | PPARda | XM_048254268 |
| | | PPARdb | XM_048240774 |
| | Denticeps clupeoides | PPARd | M_028994977 |
| | Engraulis encrasicolus | PPARdb | XM_063209213 |
| Cypriniformes | Onychostoma macrolepis | PPARda | XM_058761917 |
| | | PPARdb | XM_058783926 |
| | Carassius carassius | PPARd- like | XM_059544129 |
| | | PPARdb | XM_059503700 |
| | Carassius gibelio | PPARda | XM_052590020 |
| | | PPARdb | XM_052563340 |
| | Sinocyclocheilus rhinocerous | PPARda | XM_016566200 |
| | Labeo rohita | PPARda | XM_051105308 |
| | | PPARdb | XM_051116882 |
| | Xyrauchen texanus | PPARda | XM_052121745 |
| | | PPARd- like | XM_052109018 |
| | Myxocyprinus asiaticus | PPARda | XM_051695219 |
| | Danio rerio | PPARda | XM_694808 |
| | | PPARdb | BC162174 |
| | Danio aesculapii | PPARda | XM_056447660 |
| | | PPARdb | XM_056463648 |
| | Triplophysa dalaica | PPARda | XM_056758523 |
| | | PPARdb | XM_056773304 |
| | Triplophysa rosa | PPARda | XM_057325943 |
| | | PPARdb | XM_057356537 |
| | Ctenopharyngodon idella | PPARda | XM_051908671 |
| | | PPARdb | XM_051903590 |
| | Misgurnus anguillicaudatus | PPARda | XM_055217442 |
| | | PPARdb | XM_055167408 |
| | Rhinichthysklamathensis goyatoka | PPARda | XM_056237906.1 |
| | | PPARdb | XM_056261030 |

**PPARδ cont'd**

| | | | |
|---|---|---|---|
| Cyprinodontiformes | Poeciliopsis prolifica | PPARdb | XM_055049611 |
| | Gambusia affinis | PPARdb | XM_044127735 |
| | Xiphophorus maculatus | PPARd | XM_005801352 |
| | Xiphophorus couchianus | PPARd | XM_028022006 |
| | Xiphophorus hellerii | PPARd | XM_032573390 |
| | Poecilia formosa | PPARd | XM_007556709 |
| | Poecilia reticulata | PPARd | XM_008413546 |
| | Poecilia mexicana | PPARd | XM_015009451 |
| | Poecilia latipinna | PPARd | XM_015053582 |
| | Austrofundulus limnaeus | PPARd | XM_014019613 |
| | Kryptolebias marmoratus | PPARdb | XM_017430938 |
| | Nematolebias whitei | PPARdb | XM_037684887 |
| | Cyprinodon tularosa | PPARdb | XM_038283008 |
| | Cyprinodon variegatus | PPARd | XM_015391146 |
| | Nothobranchius furzeri | PPARdb | XM_015967772 |
| | Girardinichthys multiradiatus | PPARdb | XM_047364303 |
| | Fundulus heteroclitus | PPARdb | XM_012870585 |
| Esociformes | Esox lucius | PPARda | XM_013138276 |
| | | PPARdb | M_029123950 |
| Gadiformes | Gadus morhua | PPARd | XM_030352117 |
| | Gadus chalcogrammus | PPARdb | XM_056599370 |
| | Gadus macrocephalus | PPARdb | XM_060050977 |
| Gobiiformes | Boleophthalmus pectinirostris | PPARdb | XM_020929921 |
| Gymnotiformes | Electrophorus electricus | PPARda | M_027032363 |
| | | PPARdb | XM_027033113 |
| Holocentiformes | Myripristis murdjan | PPARd | XM_030056846 |
| Istiophoriformes | Xiphias gladius | PPARdb | XM_040152830 |
| Kurtiformes | Sphaeramia orbicularis | PPARd | XM_030139836 |
| Labriformes | Labrus bergylta | PPARd | XM_020648279 |
| | Notolabrus celidotus | PPARdb | XM_034695465 |
| | Cheilinus undulatus | PPARdb | XM_041798839 |
| | Labrus mixtus | PPARdb | XM_061058616 |
| Lampriformes | Lampris incognitus | PPARdb | XM_056275159 |
| Mugiliformes | Mugil cephalus | PPARdb | XM_047593288 |
| Osmeriformes | Hypomesus transpacificus | PPARdb | XM_047039898 |
| | Osmerus eperlanus | PPARda | XM_062486690 |
| | | PPARdb | XM_062458650 |
| Osteoglossiformes | Scleropages formosus | PPARd | XM_018746817 |
| | Paramormyrops kingsleyae | PPARd | XM_023793552 |
| | Brienomyrus brachyistius | PPARdb | XM_049002999 |
| Ovalentaria | Acanthochromis polyacanthus | PPARdb | XM_022203531 |
| | Amphiprion ocellaris | PPARdb | XM_023267792 |
| | Parambassis ranga | PPARdb | XM_028409057 |

**PPARδ cont'd**

| | | | |
|---|---|---|---|
| Perciformes | Etheostoma cragini | PPARdb | XM_034877904 |
| | Sander lucioperca | PPARdb | XM_031284457 |
| | Perca flavescens | PPARd | XM_028582553 |
| | Perca fluviatilis | PPARdb | XM_039801190 |
| | Stegastes partitus | PPARd | XM_008296520 |
| | Centropristis striata | PPARdb | XM_059332412 |
| | Epinephelus lanceolatus | PPARdb | XM_033629083 |
| | Plectropomus leopardus | PPARdb | XM_042491159 |
| | Epinephelus fuscoguttatus | PPARdb | XM_049580153 |
| | Epinephelus moara | PPARdb | XM_050064183 |
| | Trematomus bernacchii | PPARdb | XM_034144694 |
| | Notothenia coriiceps | PPARd | XM_010784397 |
| | Dicentrarchus labrax | PPARdb | XM_051425177 |
| | Morone saxatilis | PPARdb | XM_035656599 |
| | Pungitius pungitius | PPARdb | XM_037478130 |
| | Gasterosteus aculeatus | PPARdb | XM_040193242 |
| | Sebastes umbrosus | PPARdb | XM_037772750 |
| | Cottoperca gobio | PPARd | XM_029435102 |
| | Anarrhichthys ocellatus | PPARd | XM_031845071 |
| | Pseudochaenichthys georgianus | PPARdb | XM_034087402 |
| | Gymnodraco acuticeps | PPARdb | XM_034208892 |
| | Cyclopterus lumpus | PPARdb | XM_034536240 |
| | Anoplopoma fimbria | PPARdb | XM_054609340 |
| | Pseudoliparis swirei | PPARdb | XM_056422417 |
| | Larimichthys crocea | PPARdb | NM_001303386 |
| Pleuronectiformes | Pleuronectes platessa | PPARdb | XM_053423917 |
| | Limanda limanda | PPARdb | XM_061073977 |
| | Hippoglossus hippoglossus | PPARdb | XM_034589744 |
| | Hippoglossus stenolepis | PPARdb | XM_035158970 |
| | Platichthys flesus | PPARdb | XM_062392970 |
| | Solea senegalensis | PPARdb | XM_044038096 |
| | Solea solea | PPARdb | XM_058642776 |
| | Cynoglossus semilaevis | PPARd | XM_008318189 |
| | Paralichthys olivaceus | PPARd | XM_020089214 |
| | Scophthalmus maximus | PPARdb | XM_035643796 |
| Polypteriformes | Polypterus senegalus | PPARdb | XM_039748634.1 |
| Salmoniformes | Salmo trutta | PPARdb | XM_029718543 |
| | Oncorhynchus keta | PPARdb | XM_035739374 |
| | Salmo salar | PPARdb | NM_001123559 |
| | | PPARb2B | AM229306 |
| | | PPARb1A | NM_001123635 |
| Scombriformes | Scomber japonicus | PPARdb | XM_053316856 |
| | Scomber scombrus | PPARdb | XM_062427831 |

**PPARδ cont'd**

| | | | |
|---|---|---|---|
| Siluriformes | Ictalurus punctatus | PPARda | XM_017496622 |
| | | PPARdb | XM_017468665 |
| | Ictalurus furcatus | PPARda | XM_053653161 |
| | | PPARdb | XM_053625118 |
| | Pangasianodon hypophthalmus | PPARda | XM_034314382 |
| | | PPARdb | XM_026919752 |
| | Clarias gariepinus | PPARda | XM_053483984 |
| | | PPARdb | XM_053503473 |
| | Hemibagrus wyckioides | PPARda | XM_058372999 |
| | | PPARdb | XM_058389829 |
| | Tachysurus vachellii | PPARdb | XM_060880701 |
| | Neoarius graeffei | PPARda | XM_060910427 |
| | | PPARdb | XM_060931987 |
| Spariformes | Sparus aurata | PPARd | M_030423544 |
| Synbranchiformes | Monopterus albus | PPARd | XM_020618507 |
| | Mastacembelus armatus | PPARd | XM_026332476 |
| Syngnathiformes | Doryrhamphus excisus | PPARdb | XM_058083412 |
| | Phycodurus eques | PPARdb | XM_061693205 |
| | Phyllopteryx taeniolatus | PPARdb | XM_061800339 |
| | Syngnathoides biaculeatus | PPARdb | XM_061805211 |
| | Dunckerocampus dactyliophorus | PPARdb | XM_054783662 |
| | Corythoichthys intestinalis | PPARdb | XM_057829497 |
| | Nerophis ophidion | PPARdb | XM_061903501 |
| | Entelurus aequoreus | PPARdb | XM_062052906 |
| | Hippocampus comes | PPARd | XM_019878424 |
| | Hippocampus zosterae | PPARdb | XM_052052945 |
| | Syngnathus scovelli | PPARdb | XM_049754708 |
| | Syngnathus typhle | PPARdb | XM_061271871 |
| | Synchiropus splendidus | PPARdb | XM_053850474 |
| Tetraodontiformes | Takifugu rubripes | PPARdb | NM_001097628 |
| | Takifugu flavidus | PPARdb | XM_057040428 |

# PPARγ Sequences

| ORDER | Species | Isotype | GenBank Access. no |
|---|---|---|---|
| Acipenseriformes | Acipenser ruthenus | PPARγ-like | XM_058999826.1 |
| Anabantiformes | Anabas testudineus | PPARγ | XM_026350118.2 |
| | Betta splendens | PPARγ | XM_029151588.2 |
| | Scatophagus argus | PPARγ | XM_046383318.1 |
| Anguilliformes | Anguilla anguilla | PPARγ | XM_035387036.1 |
| | Conger conger | PPARγ | XM_061218876.1 |
| Atheriniformes | Melanotaenia boesemani | PPARγ | XM_041979457.1 |
| Batrachoidiformes | Thalassophryne amazonica | PPARγ | XM_034166205.1 |
| Beloniformes | Oryzias latipes | PPARγ | XM_023954652.1 |
| | Oryzias melastigma | PPARγ | XM_024269321.2 |
| | Cololabis saira | PPARγ | XM_061727404.1 |
| Blenniiformes | Gouania willdenowi | PPARγ | XM_028447341.1 |
| | Salarias fasciatus | PPARγ | XM_030091775.1 |
| Carangaria incertae sedis | Lates calcarifer | PPARγ | XM_018686891.2 |
| | Toxotes jaculatrix | PPARγ | XM_041035091.1 |
| Carangiformes | Seriola aureovittata | PPARγ | XM_056396553.1 |
| | Seriola dumerili | PPARγ | XM_022748373.1 |
| | Seriola lalandi | PPARγ | XM_023426826.1 |
| | Echeneis naucrates | PPARγ | XM_029503269.1 |
| Centrarchiformes | Siniperca chuatsi | PPARγ | XM_044220995.1 |
| | Micropterus dolomieu | PPARγ | XM_046076317.1 |
| | Micropterus salmoides | PPARγ | XM_038695875.1 |
| Chaetodontiformes | Chelmon rostratus | PPARγ | XM_041955638.1 |
| Characiformes | Astyanax mexicanus | PPARγa | XM_007229633.4 |
| | | PPARγb | XM_049463146 |
| | Pygocentrus nattereri | PPARγa | XM_017704774.2 |
| | Colossoma macropomum | PPARγa | XM_036559639.1 |
| Cichliformes | Oreochromis niloticus | PPARγ | XM_005459020.3 |
| | Maylandia zebra | PPARγ | XM_004548565.4 |
| | Pundamilia nyererei | PPARγ | XM_005724654.1 |
| | Haplochromis burtoni | PPARγ | XM_005932305.3 |
| | Neolamprologus brichardi | PPARγ | XM_006783251.2 |
| | Astatotilapia calliptera | PPARγ | XM_026167696.1 |
| | Archocentrus centrarchus | PPARγ | XM_030728757.1 |
| | Oreochromis aureus | PPARγ | XM_031731392.2 |
| | Simochromis diagramma | PPARγ | XM_040052233.1 |
| Clupeiformes | Sardina pilchardus | PPARγa | XM_062544753 |
| | | PPARγb | XM_062540999.1 |
| | Clupea harengus | PPARγa | XM_012833107.3 |
| | | PPARγb | XM_012827412 |
| | Denticeps clupeoides | PPARγa | XM_028997557.1 |
| | Alosa sapidissima | PPARγa | XM_042089567.1 |
| | Alosa alosa | PPARγa | XM_048254946.1 |
| Cypriniformes | Sinocyclocheilus grahami | PPARγ | XM_016249326.1 |
| | Sinocyclocheilus anshuiensis | PPARγ | XM_016484590.1 |
| | Sinocyclocheilus rhinocerous | PPARγ | XM_016575636.1 |
| | Cyprinus carpio | PPARγ | XM_042765856.1 |
| | Carassius auratus | PPARγ | XM_026220745.1 |
| | Carassius gibelio | PPARγ | XM_052610475.1 |
| | Labeo rohita | PPARγ | XM_051122581 |
| | Onychostoma macrolepis | PPARγ | XM_058791253.1 |
| | Carassius carassius | PPARγ | XM_059528909.1 |
| | Puntigrus tetrazona | PPARγ | XM_043251602.1 |
| | Megalobrama amblycephala | PPARγ | XM_048173304.1 |
| | Ctenopharyngodon idella | PPARγ | XM_051913344.1 |
| | Pimephales promelas | PPARγ | XM_039648555.1 |
| | Rhinichthys klamathensis goyatoka | PPARγ | XM_056266556.1 |
| | Danio rerio | PPARγ | NM_131467.1 |
| | Danio aesculapii | PPARγ | XM_056468577.1 |
| | Triplophysa dalaica | PPARγ | XM_056734291.1 |
| | Triplophysa rosa | PPARγ | XM_057361730.1 |
| | Misgurnus anguillicaudatus | PPARγ | XM_055183751.1 |
| | Xyrauchen texanus | PPARγ | XM_052111120.1 |

**PPARγ cont'd.**

| | | | |
|---|---|---|---|
| Cyprinodontiformes | Poeciliopsis prolifica | PPARγ | XM_055026974.1 |
| | Gambusia affinis | PPARγ | XM_044123478.1 |
| | Xiphophorus couchianus | PPARγ | XM_028003932.1 |
| | Xiphophorus hellerii | PPARγ | XM_032550507.1 |
| | Xiphophorus maculatus | PPARγ | XM_023325414.1 |
| | Poecilia formosa | PPARγ | XM_007549484.2 |
| | Poecilia reticulata | PPARγ | XM_008408552.2 |
| | Poecilia mexicana | PPARγ | XM_014997022.1 |
| | Poecilia latipinna | PPARγ | XM_015044448.1 |
| | Austrofundulus limnaeus | PPARγ | XM_014002837.1 |
| | Kryptolebias marmoratus | PPARγ | XM_017408856.3 |
| | Nematolebias whitei | PPARγ | XM_037678773.1 |
| | Cyprinodon variegatus | PPARγ | XM_015379176.1 |
| | Cyprinodon tularosa | PPARγ | XM_038307855.1 |
| | Nothobranchius furzeri | PPARγ | XM_015959070.2 |
| | Fundulus heteroclitus | PPARγ | XM_012881380.3 |
| | Girardinichthys multiradiatus | PPARγ | XM_047347667.1 |
| Elopiformes | Megalops cyprinoides | PPARγ | XM_036531034.1 |
| Esociformes | Esox lucius | PPARγa | XM_034297489.1 |
| | | PPARγb | XM_010902324 |
| Gadiformes | Gadus morhua | PPARγ | XM_030374406.1 |
| | Gadus chalcogrammus | PPARγ | XM_056605761.1 |
| | Gadus macrocephalus | PPARγ | XM_060069785.1 |
| Gobiiformes | Boleophthalmus pectinirostris | PPARγ | XM_020933906.2 |
| | Periophthalmus magnuspinnatus | PPARγ | XM_033966268.2 |
| Gonorynchiformes | Chanos chanos | PPARγ | XM_030777862.1 |
| Gymnotiformes | Electrophorus electricus | PPARγ | XM_027029335.2 |
| Holocentriformes | Myripristis murdjan | PPARγ | XM_030052636.1 |
| Istiophoriformes | Xiphias gladius | PPARγ | XM_040158739.1 |
| Kurtiformes | Sphaeramia orbicularis | PPARγ | XM_030135115.1 |
| Labriformes | Labrus bergylta | PPARγ | XM_020628978.2 |
| | Notolabrus celidotus | PPARγ | XM_034676021.1 |
| | Cheilinus undulatus | PPARγ | XM_041782386.1 |
| | Labrus mixtus | PPARγ | XM_061043151.1 |
| Lampriformes | Lampris incognitus | PPARγ | XM_056273873.1 |
| Mugiliformes | Mugil cephalus | PPARγ | XM_047583091.1 |
| Osmeriformes | Hypomesus transpacificus | PPARγa | XM_047025274.1 |
| | Osmerus eperlanus | PPARγb | XM_062459215 |
| | | PPARγa | XM_062459587 |
| Osteoglossiformes | Scleropages formosus | PPARγ | XM_018744573.2 |
| | Paramormyrops kingsleyae | PPARγ | XM_023799720.1 |
| | Brienomyrus brachyistius | PPARγ | XM_049022110.1 |
| Ovalentaria | Acanthochromis polyacanthus | PPARγ | XM_051947785.1 |
| | Amphiprion ocellaris | PPARγ | XM_023264231.3 |
| | Parambassis ranga | PPARγ | XM_028404964.1 |
| Pleuronectiformes | Pleuronectes platessa | PPARγ | XM_053433420.1 |
| | Hippoglossus hippoglossus | PPARγ | XM_034584288.1 |
| | Hippoglossus stenolepis | PPARγ | XM_035152543.1 |
| | Solea senegalensis | PPARγ | XM_044024557.1 |
| | Solea solea | PPARγ | XM_058632168.1 |
| | Cynoglossus semilaevis | PPARγ | XM_025059815.1 |
| | Paralichthys olivaceus | PPARγ | XM_020096840.1 |
| | Scophthalmus maximus | PPARγ | XM_035631101.2 |
| | Limanda limanda | PPARγ | XM_061070639.1 |
| Polypteriformes | Polypterus senegalus | PPARγ | XM_039770885.1 |
| Salmoniformes | Salmo salar | PPARγa | XM_014168482.2 |
| | | PPARγb | NM_001123546 |
| | Salmo trutta | PPARγa | XM_029692695.1 |
| | Oncorhynchus tshawytscha | PPARγa | XM_042306113.1 |
| | Oncorhynchus keta | PPARγa | XM_052501281.1 |
| | | PPARγb1 | XM_052481996 |
| | | PPARγb2 | XM_052483128 |
| | Oncorhynchus gorbuscha | PPARγa | XM_046338119.1 |
| | Oncorhynchus kisutch | PPARγa | XM_020484043.2 |
| | Oncorhynchus mykiss | PPARγa | XM_036984365.1 |
| | | PPARγb | NM_001197212 |
| | Salvelinus alpinus | PPARγa | XM_024136769.1 |
| | | PPARγb | XM_023990671 |
| | Salvelinus namaycush | PPARγa | XM_038983283.1 |
| | Salvelinus fontinalis | PPARγa | XM_055870006.1 |

**PPARγ cont'd.**

| | | | |
|---|---|---|---|
| Perciformes | Perca fluviatilis | PPARγ | XM_039797969.1 |
| | Etheostoma cragini | PPARγ | XM_034869704.1 |
| | Etheostoma spectabile | PPARγ | XM_032513569.1 |
| | Perca flavescens | PPARγ | XM_028576507.1 |
| | Sander lucioperca | PPARγ | XM_031301651.2 |
| | Centropristis striata | PPARγ | XM_059327399.1 |
| | Epinephelus lanceolatus | PPARγ | XM_033627223.1 |
| | Plectropomus leopardus | PPARγ | XM_042506427.1 |
| | Epinephelus fuscoguttatus | PPARγ | XM_049583111.1 |
| | Epinephelus moara | PPARγ | XM_050037490.1 |
| | Pungitius pungitius | PPARγ | XM_037490410.1 |
| | Gasterosteus aculeatus aculeatus | PPARγ | XM_040204343.1 |
| | Morone saxatilis | PPARγ | XM_035661254.1 |
| | Dicentrarchus labrax | PPARγ | XM_051415454.1 |
| | Notothenia coriiceps | PPARγ | XM_010773488.1 |
| | Trematomus bernacchii | PPARγ | XM_034138944.1 |
| | Stegastes partitus | PPARγ | XM_008289896.1 |
| | Larimichthys crocea | PPARγ | XM_010731330.3 |
| | Cottoperca gobio | PPARγ | XM_029431820.1 |
| | Anarrhichthys ocellatus | PPARγ | XM_031839594.1 |
| | Pseudochaenichthys georgianus | PPARγ | XM_034083220.1 |
| | Gymnodraco acuticeps | PPARγ | XM_034219177.1 |
| | Cyclopterus lumpus | PPARγ | XM_034531796.1 |
| | Sebastes umbrosus | PPARγ | XM_037781971.1 |
| | Anoplopoma fimbria | PPARγ | XM_054617781.1 |
| | Pseudoliparis swirei | PPARγ | XM_056438485.1 |
| | | | |
| Scombriformes | Thunnus maccoyii | PPARγ | XM_042402801.1 |
| | Thunnus albacares | PPARγ | XM_044348177.1 |
| | Scomber japonicus | PPARγ | XM_053315069.1 |
| | Scomber scombrus | PPARγ | XM_062416427 |
| Semionotiformes | Lepisosteus oculatus | PPARγ | XM_006631031.2 |
| Siluriformes | Ictalurus furcatus | PPARγa | XM_053653619.1 |
| | | PPARγb | XM_053643773 |
| | Ictalurus punctatus | PPARγa | XM_017450361.3 |
| | | PPARγb | XM_017487917 |
| | Hemibagrus wyckioides | PPARγa | XM_058373182.1 |
| | Tachysurus vachellii | PPARγa | XM_060857514.1 |
| | Tachysurus fulvidraco | PPARγa | XM_027140763.2 |
| | | PPARγb | XM_027159460.2 |
| | Pangasianodon hypophthalmus | PPARγa | XM_026932668.3 |
| | | PPARγb | XM_026945021 |
| | Silurus meridionalis | PPARγa | XM_046875056.1 |
| | Clarias gariepinus | PPARγb | XM_053483724.1 |
| | Neoarius graeffei | PPARγa | XM_060910258.1 |
| Spariformes | Sparus aurata | PPARγ | XM_030421186.1 |
| | Acanthopagrus latus | PPARγ | XM_037100872.1 |
| Synbranchiformes | Monopterus albus | PPARγ | XM_020609689.1 |
| | Mastacembelus armatus | PPARγ | XM_026305844.1 |
| Syngnathiformes | Hippocampus comes | PPARγ | XM_019892497.1 |
| | Hippocampus zosterae | PPARγ | XM_052076498.1 |
| | Syngnathus acus | PPARγ | XM_037251615.1 |
| | Syngnathus scovelli | PPARγ | XM_049733984.1 |
| | Syngnathus typhle | PPARγ | XM_061291114.1 |
| | Dunckerocampus dactyliophorus | PPARγ | XM_054783927.1 |
| | Corythoichthys intestinalis | PPARγ | XM_057845112.1 |
| | Doryrhamphus excisus | PPARγ | XM_058054674.1 |
| | Phycodurus eques | PPARγ | XM_061686942.1 |
| | Phyllopteryx taeniolatus | PPARγ | XM_061784461.1 |
| | Syngnathoides biaculeatus | PPARγ | XM_061831841.1 |
| | Synchiropus splendidus | PPARγ | XM_053866962.1 |
| Tetraodontiformes | Takifugu rubripes | PPARγ | XM_029825888.1 |
| | Takifugu flavidus | PPARγ | XM_057029093.1 |
