# Supplementary material for: The Peroxisome Proliferator-Activated Receptors of Ray-Finned Fish: Unique Structures, Elusive Functions
Source: Biomolecules. 2024 May 29;14(6):634. doi: 10.3390/biom14060634 (PMC11201486; doi:10.3390/biom14060634)
Supplement: Supplementary file 1 [file biomolecules-14-00634-s001.zip › Table S2.pdf]

Table S2. Residues defining the ligand binding cavity of huma, lungfish coelacanth and chondrichthyan PPARγ

	hPPARα	lungfish	Coelacanth	Chondr.	hPPARδ	lungfish	Chondr.	hPPARγ	lungfish	Coelacanth	Chondr.
Arm I	F273	F	F	F	F245	F	F	F282	F	F	F
	C276	C	C	C	C249	C	C	C285	C	C	C
	Q277	Q	Q	Q	Q250	Q	Q	Q286	Q	Q	Q
	**S280**	S	S	S	**T253**	S	T	**S289**	S	S	S
	**Y314**	Y	Y	Y	**H287**	H	H	**H323**	H	H	H
	I317	M	M	M	I290	I	I	I326	I	I	I
	F318	F	F	F	F291	F	F	Y327	F	I	F
	I354	I	I	I	I327	I	I	F363	F	M	I
	**H440**	H	H	H	**H413**	H	H/N	**H449**	H	H	H
	V444	V	V	V	M417	I	V	L453	L	L	L
	L460	L	L	L	L433	L	L	L469	L	L	L
	**Y464**	Y	Y	Y	**Y437**	Y	Y	**Y473**	I	Y	Y
Arm II	I241	I	I	I	I213	I	I	I249	I	I	I
	L247	L	L	L	L219	L	L	L255	L	L	L
	E251	E	E	E	E223	E	E	E259	D	D	E
	L254	L	L	L	W228	W	W	F264	Y	Y	F
	I272	I	I	I	V245	V	V	I281	I	I	I
	C275	C	C	R	R248	R	R	G284	R	R	R
	M330	L	L	M	L303	L	L	V339	L	L	L
	V332	V	V	V	V341	V	V	I341	I	I	I
	I339	I	I	I	V348	V	V	M348	M	M	M
	F343	F	F	F	F305	F	F	F352	F	F	F
	L344	L	L	L	L317	L	L	L353	L	L	L
	M355	M	M	M	I328	M	M	M364	K	M	M
ENTRANCE	N219				N191			P227			
	M220				M192			L228			
	T279	T	T	T	T252	T	T	R288	R	R	R
	E282	E	E	E	E255	E	E	E291	E	E	E
	T283	T	T	T	T256	T	T	A292	A	A	A
	E286	E	E	E	E259	E	E	E295	E	E	E
	L321	L	L	L	L294	L	L	L330	L	L	L
	V324	I	V	I	I297	I	I	L333	L	L	L
	A333	A	A	A	A306	A	A	S342	A	A	A
	Y334	Y	Y	Y	N307	S	N	E343	N	N	A
C. C.	K292	K	K	K	K265	K	K	K301	K	K	K
	E462	E	E	E	E435	E	E	E471	E	E	E
